# Azithromycin ameliorated cigarette smoke-induced airway epithelial barrier dysfunction by activating Nrf2/GCL/GSH signaling pathway

**DOI:** 10.1186/s12931-023-02375-9

**Published:** 2023-03-06

**Authors:** Yun Song, Wenhuan Fu, Youzhi Zhang, Doudou Huang, Jian Wu, Shuangmei Tong, Mingkang Zhong, Huifang Cao, Bin Wang

**Affiliations:** 1 Department of Pharmacy, Huashan Hospital, Fudan University, Shanghai, 200040 China; 2 Department of Respiration, Huashan Hospital, Fudan University, Shanghai, 200040 China; 3 Institute of Chinese Materia Medica, Shanghai University of Traditional Chinese Medicine, Shanghai, 201203 China; 4 Department of Respiratory and Critical Medicine, Jing’an District Centre Hospital of Shanghai (Huashan Hospital Fudan University Jing’an Branch), Shanghai, 200040 China

**Keywords:** Airway epithelial barrier dysfunction, GSH metabolism, Nrf2, Azithromycin, Cigarette smoke

## Abstract

**Background:**

Airway epithelium is the first barrier against environmental insults, and epithelial barrier dysfunction caused by cigarette smoke (CS) is particularly relevant to chronic obstructive pulmonary disease (COPD) progression. Our study was to determine whether Azithromycin (AZI) ameliorates CS-induced airway epithelial barrier dysfunction and the underlying mechanisms.

**Methods:**

Primary bronchial epithelial cells (PBECs), human bronchial epithelial cells (HBECs), Sprague Dawley rats and nuclear factor erythroid 2-related factor 2 (Nrf2)−/− mice were pretreated with AZI and subsequently exposed to CS. Transepithelial electronic resistance (TEER), junction proteins as well as pro-inflammatory cytokines and apoptosis markers were examined to assess epithelial barrier dysfunction. Metabolomics study was applied to explore the underlying mechanism of AZI.

**Results:**

CS-induced TEER decline and intercellular junction destruction, accompanied with inflammatory response and cell apoptosis in PBECs were restored by AZI dose-dependently, which were also observed in CS-exposed rats. Mechanistically, GSH metabolism pathway was identified as the top differentially impacted pathway and AZI treatment upregulated the activities of glutamate cysteine ligase (GCL) and the contents of metabolites in GSH metabolic pathway. Furthermore, AZI apparently reversed CS-induced Nrf2 suppression, and similar effects on airway epithelial barrier dysfunction were also found for Nrf2 agonist tert-butylhydroquinone and vitamin C. Finally, deletion of Nrf2 in both HBECs and C57BL/6N mice aggravated CS-induced GSH metabolism imbalance to disrupt airway epithelial barrier and partially deprived the effects of AZI.

**Conclusion:**

These findings suggest that the clinical benefits of AZI for COPD management are related with the protection of CS-induced airway epithelial barrier dysfunction via activating Nrf2/GCL/GSH pathway, providing potential therapeutic strategies for COPD.

**Graphical abstract:**

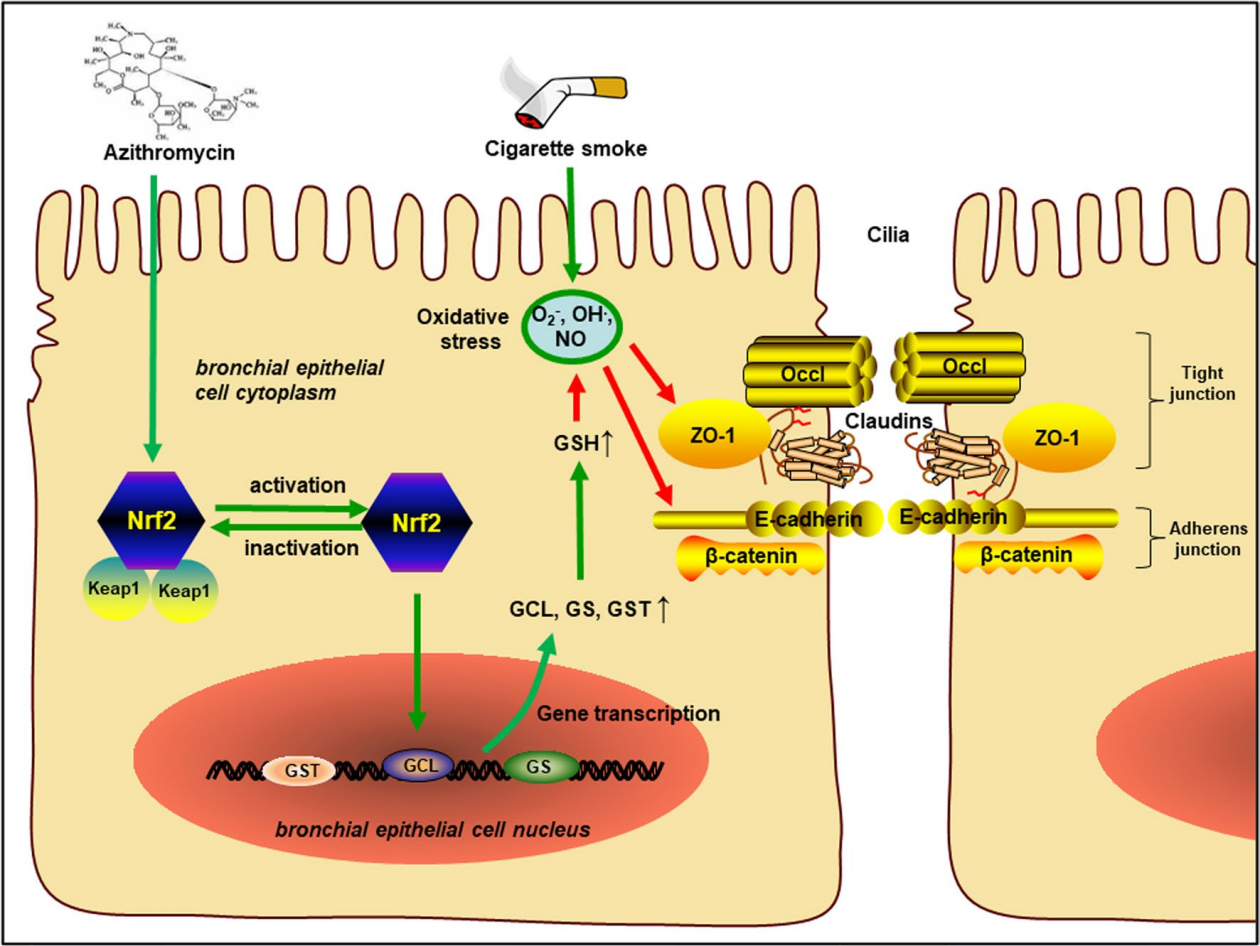

**Supplementary Information:**

The online version contains supplementary material available at 10.1186/s12931-023-02375-9.

## Background

Chronic obstructive pulmonary disease (COPD) is a respiratory disease characterized by persistent and irreversible airflow restriction, which is related with abnormal airway inflammatory response to harmful gases/particles [1]. Currently, COPD is a global chronic disease with high morbidity, mortality and disability rate, and has become an important public health problem in the world [2]. Smoking is the major risk factor for COPD, and cigarette smoke (CS)-induced oxidative stress can lead to the damage of airway epithelium and compromise the barrier function, which is the initial step and pathological basis of COPD progression [3–6].

Airway epithelium is the first line of defense against inhaling particles and pathogens due to its barrier property, which plays a crucial role in regulating innate barrier immunity and maintaining homeostasis [7]. Airway epithelial barrier dysfunction not only impairs the physical barrier activity of airway epithelium, but further exposes sub-epithelial layers to exogenous substances, leading to a series of pathological processes such as airway inflammation, airway remodeling, airway hyperresponsiveness and increasing the rate of COPD exacerbation [7, 8]. The barrier function of airway epithelium is maintained by apical junctional complexes consisting of tight junction (TJ) and adherent junction (AJ). The TJ proteins, such as zonula occludens (ZO), claudin, occludin, and the AJ proteins, such as E-cadherin, jointly participate in the formation of airway epithelial barrier [5, 6, 9]. Studies have reported acute changes in airway epithelial permeability due to oxidative stress and inflammation, and junction proteins gradually decomposed after long-term CS exposure, leading to the structural destruction of airway epithelial barrier [10–12]. Clinical data also confirmed that the expressions of ZO-1, occludin, and E-cadherin was significantly down-regulated in the bronchial epithelium and lung tissues in COPD patients compared to healthy controls [5, 13, 14]. Accordingly, the structural and functional disorder of airway epithelial barrier may be a key link in the occurrence and development of COPD [5, 15].

In recent years, great progress has been made in the treatment of COPD, while current therapies are mainly palliative. It is of great significance to find novel effective therapies targeting airway epithelial barrier. The Global Initiative for Chronic Obstructive Lung Disease [16] recommended Azithromycin (AZI), a semi synthetic 15 membered macrolide antibiotic, as anti-inflammatory drug in stable COPD and for patients who still develop exacerbations on bronchodilators/inhaled glucocorticoid therapy. AZI can inhibit the synthesis of bacterial proteins by blocking the assembly of ribosomal units, and exert powerful antibacterial effects on a wide spectrum of gram-positive and gram-negative bacteria and atypical bacteria [17]. Because of the good safety, tolerance and special pharmacokinetic characteristics, it is widely used in clinical practice for various respiratory diseases. Several randomized controlled studies have shown that long-term additional treatment with AZI significantly reduced the exacerbation rate of COPD [18–20]. Besides antibacterial effect, AZI also showed antioxidant, anti-inflammatory and other pharmacological activities. For instance, AZI treatment ameliorated epithelial cell shedding after injury in addition to a dampened inflammatory response in a mouse model in a SO_2_-induced mouse model [21]. AZI up-regulated the expression of nuclear factor erythroid 2-related factor 2 (Nrf2), an anti-oxidant transcription factor, thereby suppressing inflammatory response induced by CS exposure [22]. However, the effects of AZI and its mechanisms on airway epithelial barrier dysfunction caused by CS have not yet been elucidated.

In this study, we first investigated the protective effects of AZI targeting airway epithelial barrier using in vivo and in vitro cigarette smoking models. Then we employed a LC–MS-based metabolomics approach to obtain the metabolic profiles of in vivo experimental model to evaluate the underlying mechanisms. Our results demonstrated that GSH-related Nrf2 signaling pathway can be pharmacologically manipulated by AZI, which may provide new possibilities in the treatment of airway epithelium damage-related respiratory diseases induced by CS, such as COPD.

## Methods

### Materials

Macrolides were purchased from Selleck Chemicals (Houston, TX, USA). The antibodies used in present study were listed as follows: anti-Bax (#T40051F) and anti-Bcl-2 (#T40056) antibodies were purchased from Abmart biomedical Co., Ltd (Shanghai, China). Anti-GAPDH (#97166S), anti-Nrf2 (#12721S), anti-E-Cadherin (#14472), anti-ZO-1 (#13663S) and Alexa Fluor 594/488 conjugated anti-rabbit (#8889S)/mouse (#4408S) IgG (H + L) F(ab')2 Fragments were purchased from Cell Signaling Technology (Danvers, MA, USA). Anti-Keap1 (#ab19403) antibodies were purchased from Abcam Biotechnology (Cambridge, MA, USA). The HRP-labeled goat anti-rabbit (#65-6120)/mouse (#62-6520) IgG (H + L) secondary antibodies, ECL Plus Western Blotting Detection Kit (#32209) and BCA protein assay kit (#23227) were purchased from Thermo Fisher Scientific (San Jose, CA, USA). All other reagents were purchased from Beyotime Biotechnology (Shanghai, China) or Sangon Biotech Co., Ltd. (Shanghai, China) unless otherwise indicated.

### Animal experiments and treatments

Male Sprague Dawley (SD) rats (8 weeks old, weighed 180–200 g) were purchased from SLAC Laboratory Animal Co., Ltd. (Shanghai, China). Nrf2 knockout mice (C57BL/6N background) were purchased from Cyagen Biosciences Inc (Suzhou, Jiangsu, China). The animals were housed at a constant temperature (25 ℃) under a 12 h light–dark cycle and had free access to food and water. The protocol has the approval of the Animal Experimental Ethical Committee of Fudan University (2019 Huashan Hospital JS-112) and all animal studies were performed in accordance with the guidelines for the care and use of laboratory animals set by Fudan University (Shanghai, China). The CS-induced COPD rat model was established as our previous report [23]. 30 SD rats were randomly divided into 5 groups (6 rats/ group): control group, COPD model group, AZI low-dose (25 mg/kg) group, AZI middle-dose (50 mg/kg) group and AZI high-dose (100 mg/kg) group. AZI was administered intragastrically 1 h before the first exposure every day. The CS-exposed mice model was established according to a previously described protocol [24]. Both wild type mice (n = 18) and Nrf2 (−/−) mice (n = 18) were randomly divided into 3 groups (6 mice/ group) respectively: control group, CS group and AZI-treated group (100 mg/kg). Control group was treated with saline. After the experiment, the rats or mice were anesthetized with intraperitoneal sodium pentobarbital (40 mg/kg) for sample collection and further analysis.

### Bronchoalveolar lavage fluid (BALF) preparation

After anaesthesia, tracheotomy was performed to insert the cannula into the trachea. BALF was collected from the right lungs through three lavages of 1 ml saline for rats or two lavages of 0.5 ml saline for mice. Extracted BALF was immediately centrifuged at 1000 rpm for 5 min at 4 ℃ and used for further study.

### Immunohistochemistry staining

Briefly, immediately after bronchoalveolar lavage, the right lungs were immersed in 4% paraformaldehyde for 24 h. After tissue fixation and paraffin embedding, 5 μm sections were incubated with anti-E-cadherin, ZO-1, Keap1 and Nrf2 antibodies (1:100 dilution) at 4 ℃ overnight followed by incubation with secondary antibody, at last stained with DAB and counterstained with hematoxylin.

### Cell acquisition and culture

Primary bronchial epithelial cells (PBECs) were obtained from bronchial brushings in six healthy subjects (Additional file 1: Table S1). The Medical Ethics Committee of Huashan Hospital approved the study (KY2019-508), and all subjects gave their written informed consent. After protease digestion and centrifugation (1200 rpm, 5 min), PBECs were cultured in bronchial epithelial cell medium (#3211, ScienCell, San Diego, California, USA), supplemented with 100 U/ml penicillin, 100 μg/ml streptomycin and 1 × bronchial epithelial cell growth supplement (#3262, ScienCell, San Diego, California, USA) using poly-L-lysine-coated flasks and employed for experiments at passage 2–4 without mycoplasma contamination (Additional file 2: Fig. S1). Human bronchial epithelial cells (HBECs, ZQ0001, ScienCell, San Diego, California, USA) were cultured in Keratinocyte Medium (#2111, ScienCell, San Diego, California, USA), supplemented with 100 U/ml penicillin, 100 μg/ml streptomycin and 1 × keratinocyte growth supplement (#2162, ScienCell, San Diego, California, USA). Unless otherwise stated, cell culture reagents were purchased from Gibco (Carlsbad, CA, USA).

### Cell treatment and cigarette smoke extract (CSE) preparation

Cells were pre-treated with or without AZI (0.5, 5, 50 μM), vitamin C (50 μM) or TBHQ (30 μM) for 1 h and subsequently exposed to vehicle (medium) or 3% CSE for 24 h. As our previous reports [25], CSE was prepared by the combustion of one cigarette (12 mg tar/cigarette; Double Happiness, China), using a pump and passing the smoke through 10 mL of non-FBS culture medium at a rate of 5 min/cigarette. The resulting solution was adjusted to pH 7.4 with 1.0 M NaOH and strained through 0.22 μm gauge filters. The OD value of the obtained solution was 0.2 at 408 nm by a microplate reader, which represented 100% concentration and was diluted to the desired concentration with non-FBS culture medium. The fresh CSE was used within 30 min.

### Measurement of transepithelial electrical resistance (TEER)

Airway epithelial permeability changes were evaluated by TEER measurement using a Millicell ERS-2 V-Ohmmeter (Millipore Co., Bedford, MA, USA) monitoring for 24 h at specified time points. In detail, PBEC/HBECs cells (10^5^/well) were seeded in 12-well hanging inserts (0.4 μm, PET, Cat.No: MCHT12H48, Millipore, Darmstadt, Germany) with 500 μl apical and 1000 μl basolateral volumes of complete medium. HBECs were incubated for 48 h to yield a cell monolayer. Before TEER measurement, hanging inserts were equilibrated at room temperature for 10 min. After soaking in 70% ethanol and rinsing with medium, the electrode was inserted vertically into the chamber (below the liquid level without touching the bottom). TEER was calculated by the following equation: TEER (Ω/cm^2^) = (R_sample_– R_blank_) × effective membrane area (cm^2^). TEER values were corrected for background resistance of medium without cells.

### Inflammatory cytokines and oxidative stress indexes

The levels of IL-6 and TNF-α in the BALF or culture supernatant were determined with ELISA kit (Jianglai industrial limited ByShare Ltd, Shanghai, China) according to the manufacturer's instructions. The contents of GSH and ROS, the activity of GST, GS and GCL in lung homogenate or cell lysate were detected using commercial assay kit (Sangon Biotech Co., Ltd., Shanghai, China) according to the manufacturer's instructions.

### Western blot

Total protein was extracted by the RIPA (Beyotime, Shanghai, China), separated by 8–12% SDS-PAGE and electro transferred onto a PVDF membrane (Millipore, Bedford, MA, USA). The membrane was blocked with 5% skim milk for 1 h at room temperature and incubated with primary antibodies Bax, Bcl-2, Keap1, Nrf2, ZO-1, E-cadherin (1:1000 dilution) overnight at 4 ℃, followed by HRP-conjugated secondary antibodies for additional 1 h at 37 ℃. Proteins were visualized with BIO-RAD Molecular Imager (Version 6.0, USA) using enhanced chemiluminescence reagents.

### Flow cytometry

The flow cytometry assays were performed according to manufacturer's instructions. Apoptosis was determined with the Annexin V-FITC/PI apoptosis detection kit (BD Bioscience, San Jose, CA, USA) and a flow cytometer (Beckman Coulter Inc., Brea, CA, USA).

### Immunofluorescence staining

Briefly, cells were seeded on confocal dishes and treated as indicated for 24 h. The dishes were washed three times with PBS, fixed in 4% paraformaldehyde for 20 min at 37 ℃, and then permeabilized with 0.3% Triton X-100 for 10 min. Cells were then blocked with 3% BSA at room temperature for 1 h followed by incubation with a primary antibody E-cadherin, ZO-1, Keap1 and Nrf2 (1:100 dilution) at 4 ℃ overnight. Dishes were washed three times with PBS and incubated with Alexa Fluor 594/488 conjugated-goat anti-rabbit/mouse IgG for 1 h, and then labeled with hoechst for 15 min. Finally, Dishes were washed three times with PBS, visualized and photographed under confocal laser scanning microscope (Leica TCS SP8, Germany).

### Short hairpin RNA (shRNA) interference

HBECs grown to 30–50% confluency were transfected by lentiviral-delivered shRNAs targeting Nrf2 (multiplicity of infection = 20, GENECHEM Incorporation, Shanghai, China), and simultaneously strengthened by HitransG P (1x). After 8 h transfection, cells were incubated with fresh medium for another 48 h, and subsequently screened with puromycin (2 μg/mL) for 72 h. Monoclonal cells were maintained for further experiment. The sequences of shRNA-Nrf2 are as follows: 5’-CCGGCATTTCACTAAACACAA-3’.

### Method of metabolomics detection and analysis

For lung tissue homogenate samples, 400 μl MeOH (containing 100 ng/mL Warfarin as Internal Standard) was added to the samples (100 μl), which were then vortexed for 60 s, followed by centrifugation at 14,000*g* and 4 ℃ for 15 min. Finally, the supernatants were transferred to HPLC glass vials and stored at − 20 ℃ prior to LC–MS analysis. For data acquisition, the metabolomics data was acquired by Agilent 1290 UHPLC system coupled to a quadruple time-of-flight mass spectrometer (6530 Q/TOF–MS). Analytes in samples were separated with an ACQUITY BEH C18 (2.1 × 100 mm, 1.7 μm) (Waters Technologies, Milford, MA, USA); the column temperature was 30℃. The mobile phase consisted of 0.1% formic acid in water (A) and 0.1% formic acid in acetonitrile (B), and the flow rate was 0.3 mL/min. Gradient elution was carried out as follows: 5–5% B over 0–3 min; 5–10% B over 3–3.5 min; 10–40% B over 3.5–12 min; 40–60% B over 12–22 min; 60–80% B over 22–26 min; 100–100% B over 26.3–30 min. Re-equilibration was at 5% B for 3 min. A quality control sample was employed to monitor the system stability by injecting after each 10 injection. The MS condition was set according to previous study [26]. For data processing, non-targeted LC–MS data from multiple runs were extracted and aligned using MS-DIAL software. The metabolites were identified according to MS-FINDER software based on MS1 and MS2 analysis. The raw MS data files were converted to the mzXML format using ProteoWizard, and processed using the MS-DIAL software for peak detection, alignment, and isotope annotation. After processing, a peak list containing the mass-to-charge ratio (m/z), retention time and peak intensity was generated. The peak list was imported to MetaboAnalyst 5.0 by sum, then features were calculated the *p* values using *t*-test and p < 0.05 were considered as differential metabolites, and were further identified based on the exact molecular weight and the MS/MS spectrum similarity using the MS-FINDER software according to METLIN and HMDB data. The metabolite set enrichment analysis (MSEA) of different metabolites was conducted based on online MetaboAnalyst 5.0 platform.

### Data and statistical analysis

Data analysis was performed using GraphPad Prism software (Version 7.0, La Jolla, CA, USA), and expressed as means ± SD. The *t*-test was performed to measure the differences between the two groups and ANOVA followed by a Dunnett’s test was performed to compare the differences among three or more groups. A two-sided P value less than 0.05 were considered to be significantly different.

## Results

### AZI ameliorated CS-induced airway epithelial barrier dysfunction in vitro and in vivo

**Fig. 1:**
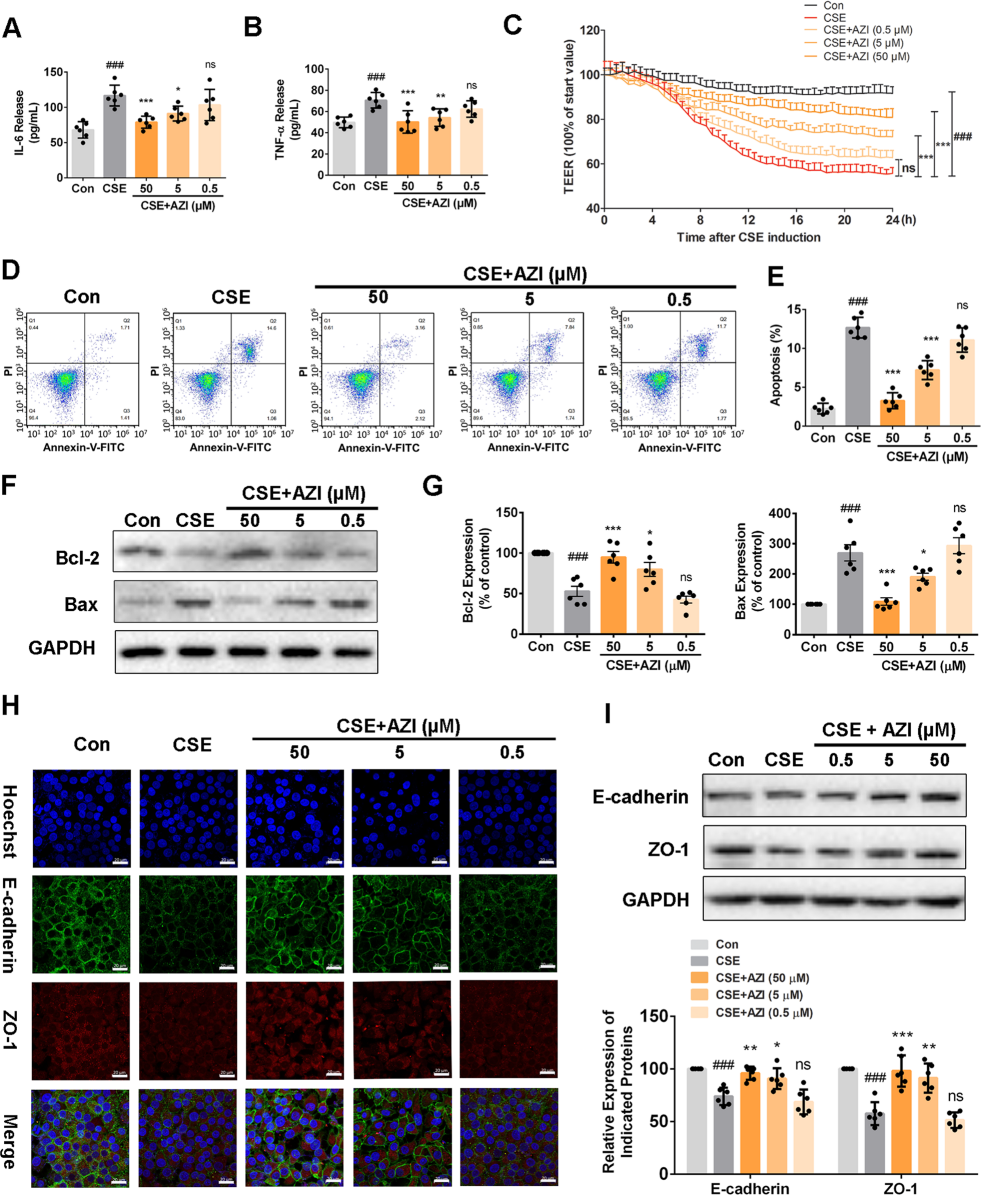
Role of AZI on airway epithelial barrier dysfunction in CSE-incubated PBECs. PBECs were pre-incubated with or without different concentrations of AZI (50, 5, 0.5 μM) for 1 h, and subsequently co-incubated with 3% CSE for 24 h. **A**, **B** The levels of IL-6 and TNF-α in the supernatant of PBECs were measured by ELISA. **C** The TEER was monitored for 24 h at specified time points in PBECs. **D**, **E** Flow cytometry assay was used to detect the apoptosis of PBECs. **F**, **G**, **I** Western blot was performed to examine the expressions of the indicated proteins in PBECs. **H** The distributions and expressions of ZO-1 and E-cadherin in PBECs were detected by immunofluorescence staining. The scale bar represents 20 μm. Data shown are means ± SD, n = 6, with 3 replicates for each sample. ^###^P < 0.001 vs control group; ^*^P < 0.05, ^**^P < 0.01, ^***^P < 0.001 and ns means no significant difference vs CSE group

Secretions of many pro-inflammatory factors induced by CS exposure impaired the physical barrier of airway epithelium, including IL-6 and TNF-α [27]. We first examined whether CS-induced inflammatory response could be attenuated by AZI treatment. After exposure to CSE for 24 h, the levels of IL-6 and TNF-α in the supernatant of PBECs were significantly increased, whereas pretreatment with AZI significantly attenuated the release of IL-6 and TNF-α in a concentration-dependent manner (Fig. 1A, B).

Next, TEER measurement was employed to assess the integrity of epithelial barrier through measuring their capacity to impede electric current applied across the epithelial layer, and apparent decrease of TEER was observed in CSE-exposed PBECs compared with normal cells, which was recovered by AZI concentration-dependently (Fig. 1C). Moreover, the results of flow cytometry assay revealed that AZI treatment could suppress the apoptosis of PBECs exposed to CSE (Fig. 1D, E). Western blot results also showed that AZI could decrease the expression of apoptosis-related marker protein Bax in CSE group compared with control. As expected, an opposite trend was found in the expression of anti-apoptotic protein Bcl-2 (Fig. 1F, G).

Structural disruptions of AJ and TJ are identified as common hallmarks of chronic inflammation, particularly in the respiratory epithelium [5]. Thirdly, structural features analysis by immunofluorescence staining and western blot revealed that oxidative stress-induced downregulations of AJ protein (E-cadherin) and TJ protein (ZO-1) were markedly reversed by AZI treatment (Fig. 1H, I). These results suggested that CSE-induced epithelial barrier dysfunction could be ameliorated by AZI in a concentration-dependent manner in vitro.

**Fig. 2:**
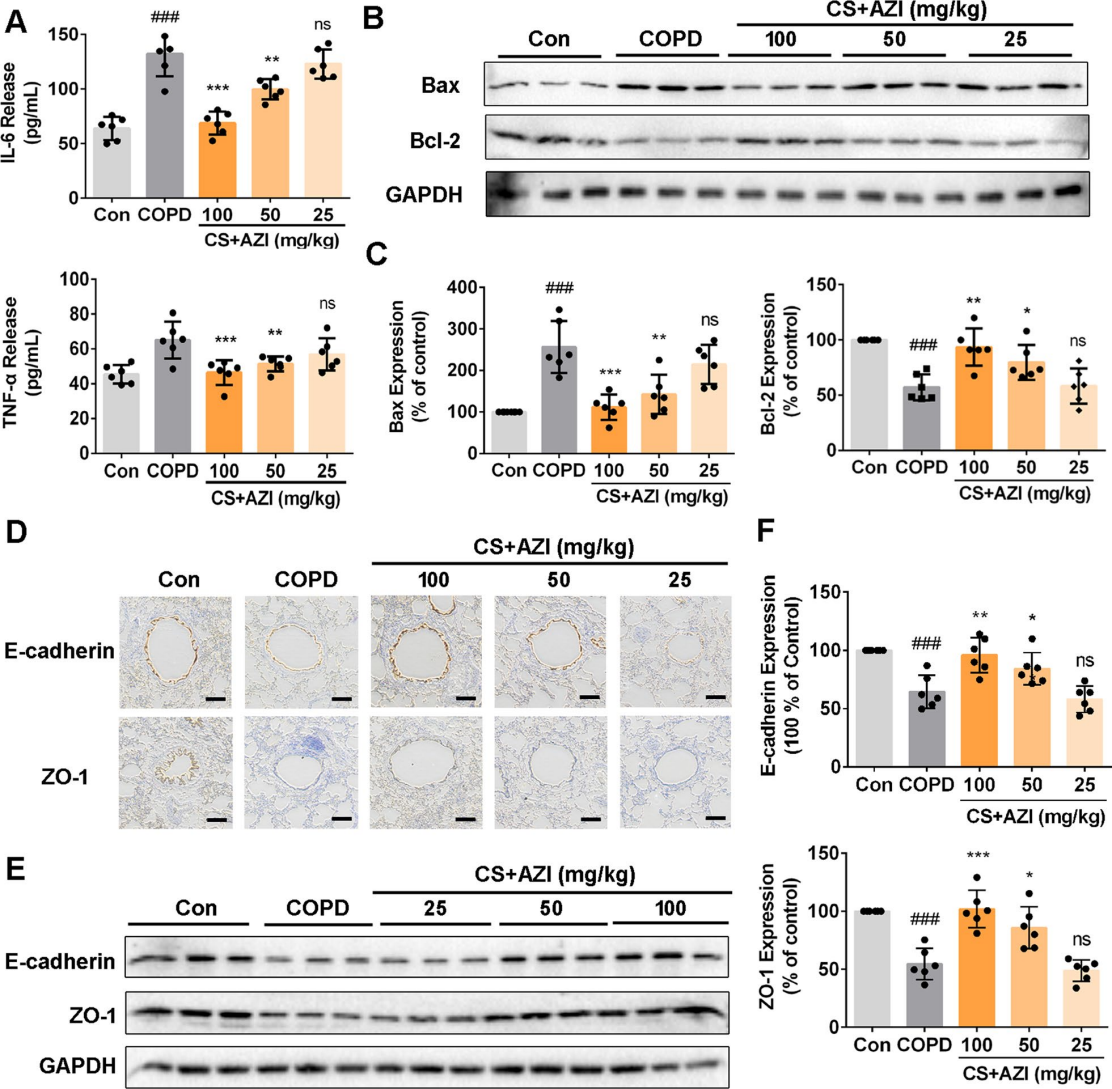
Role of AZI on airway epithelial barrier dysfunction in CS-exposed rats. After CS exposure for 12 weeks with or without AZI treatment, the BALF and lung tissues of rats were collected for further experimental studies, respectively. **A** The contents of IL-6 and TNF-α in the BALF of rats were analyzed by ELISA. **B**, **C**, **E**, **F** Western blot analysis was performed to determine the expressions of indicated proteins in the lung tissues of rats. **D** Immunohistochemical staining for ZO-1 and E-cadherin was performed in the lung tissues of rats. The scale bar represents 100 μm. Data shown are means ± SD, n = 6, with 3 replicates for each sample. ^###^P < 0.001 vs control group; ^*^P < 0.05, ^**^P < 0.01, ^***^P < 0.001 and ns means no significant difference vs COPD group

In view of the significant benefits from AZI in vitro*,* we next investigated the effects of AZI on airway epithelial barrier dysfunction in vivo*.* A COPD rat model was established after 12 weeks’ CS exposure, with characteristics of lung function decline, airway inflammation, airway remodeling, emphysema and airway hyperresponsiveness as our previous study reported [23]. In COPD rat model, we found the increase of IL-6 and TNF-α in the BALF while this inflammatory response was obviously attenuated by AZI in a dose-dependent manner (Fig. 2A). Western bolt analysis also showed the up-regulation of Bax and the down-regulation of Bcl-2 in the lung tissues of CS-exposed rats, which was significantly prevented by AZI treatment (Fig. 2B, C). Moreover, we also found that both TJ protein ZO-1 and AJ protein E-cadherin were significantly suppressed in the lung tissues of CS-exposed rats compared with sham-treated rats, and this suppression could be prevented by AZI (Fig. 2D–F). These results further confirmed that, as beneficial in vitro*,* AZI also possessed protective effects against CS-induced airway epithelial barrier dysfunction in vivo.

### AZI promoted GSH metabolism under CS exposure in vivo

**Fig. 3:**
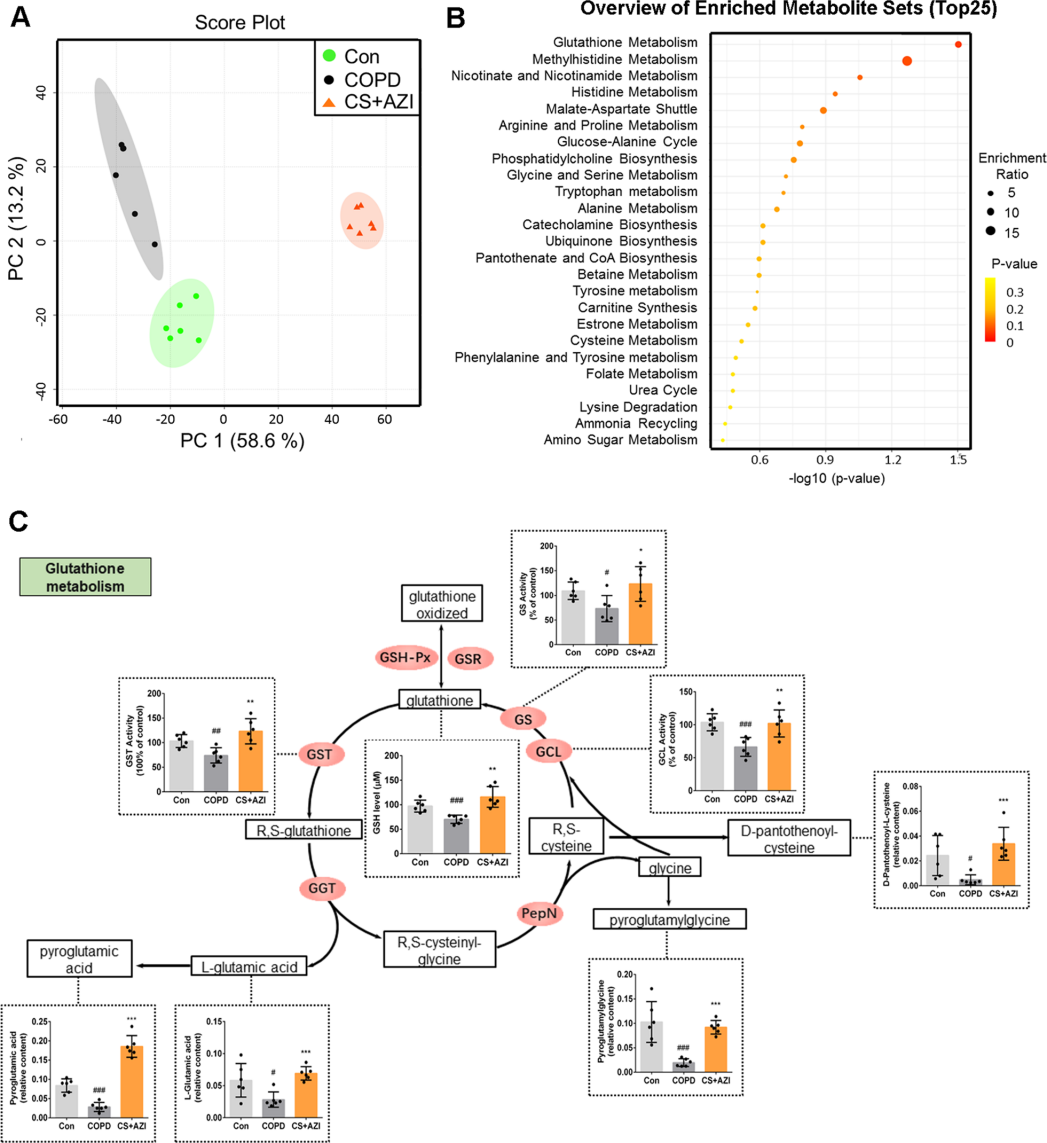
Role of AZI on GSH metabolism under CS exposure in the rat lung tissues. After12 weeks’ CS exposure with or without AZI pretreatment, the lung homogenate of SD rats was prepared for the metabolomics assay. **A** PCA of LC–MS data from control group, COPD group and CS + AZI group. **B** MSEA identified differentially regulated metabolic pathways. **C** Cartoon depicting metabolic processes, relevant metabolites and enzymes in GSH metabolism along with indicated quantitative analysis. Data shown are means ± SD, n = 6. ^#^P < 0.05, ^##^P < 0.01 and ^###^P < 0.001 vs control group; ^*^P < 0.05, ^**^P < 0.01 and ^***^P < 0.001 vs COPD group

To explore the mechanisms involved in the effects of AZI against the airway epithelial barrier dysfunction caused by CS, LC–MS was employed and 5602 metabolites were identified in the lung homogenate of SD rats. Principal component analysis (PCA) was applied to display the classification of data and obtain the intuitive distribution of samples among various groups. As shown in Fig. 3A, the PCA score plots of the samples confirmed that data of the three groups was clearly separated from each other. Here, 25 top metabolic pathways, involved in 23 differential metabolites among control group, COPD group and CS + AZI group (P < 0.05, Additional file 3: Table S2), were enriched by MSEA (Fig. 3B). GSH metabolism was identified as the top differentially impacted pathway (P < 0.05), which was finally determined as follow-up exploratory target. The further analysis confirmed a close correlation between the effects of AZI and GSH metabolism (Fig. 3C). In addition, the major metabolites and their derivatives, such as l-Glutamic acid, pyroglutamic acid, pyroglutamylglycine, d-pantothenoyl-cysteine, and the activities of some key enzymes involved in GSH metabolism pathway, such as glutamate cysteine ligase (GCL), glutathione synthetase (GS) and glutathione S-transferase (GST), were all significantly downregulated by CS exposure, while these alterations were prominently restored by AZI treatment (Fig. 3C), which was consistent with the MSEA results. Based on the above analysis, we speculated that GSH metabolism (including synthesis and consumption) might be involved in the protective effects of AZI on maintaining redox homeostasis in airway epithelial barrier dysfunction caused by CS.

### Involvement of Nrf2 in AZI-ameliorating airway epithelial barrier dysfunction in vitro and in vivo

**Fig. 4:**
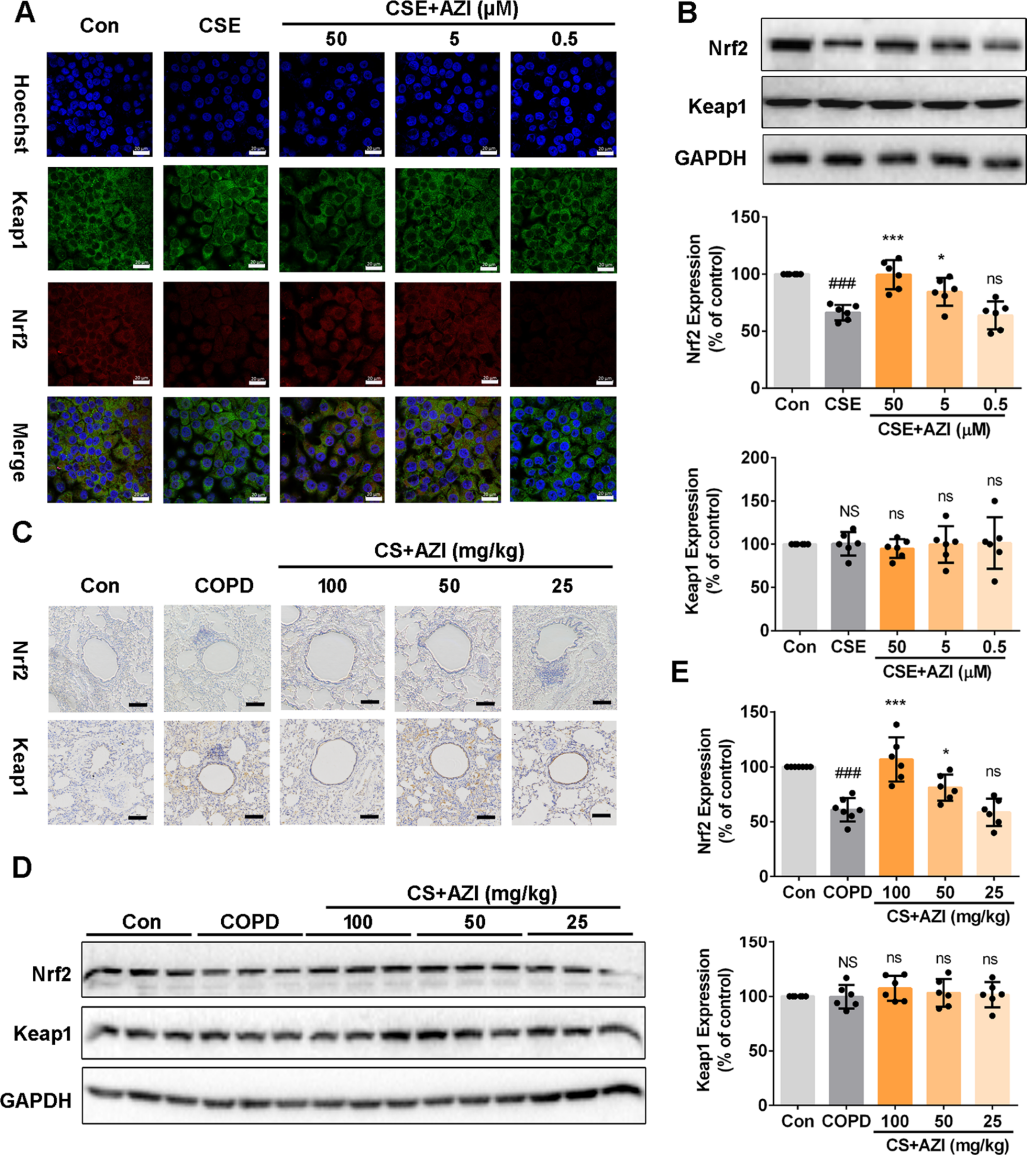
Role of AZI on Nrf2 expression in HBECs and lung tissues of CS-exposed rats. **A** Immunofluorescence staining for Nrf2 and Keap1 was performed in HBECs. The scale bar represents 20 μm. **B**, **D**, **E** Western blot analysis was used to detect Nrf2 and Keap1 expressions in HBECs and lung tissues of rats. **C** Immunohistochemical staining for Nrf2 and Keap1 was performed in the lung tissues of rats. The scale bar represents 100 μm. Data shown are means ± SD, n = 6, with 3 replicates for each sample. ^###^P < 0.001 and NS means no significant difference vs control group; ^*^P < 0.05, ^***^P < 0.001 and ns means no significant difference vs COPD/CSE group

Oxidative stress is highly correlated with the impairment of GSH metabolism in the pathogenesis of CS-induced lung diseases, such as COPD [28]. In multiple pathological processes, GSH metabolism pathway is directly regulated by Nrf2 [29, 30]. According to the results of metabolomics analysis, we subsequently investigated whether Keap1/Nrf2 signaling pathway was involved in the protective effects of AZI. Immunofluorescence staining demonstrated that Nrf2 was downregulated by CSE and recruited into the nucleus, which was restored by AZI pretreatment in a concentration-dependent manner (Fig. 4A). Similar results were also found in Western blot test (Fig. 4B). However, the expression of Keap1 was not affected by CSE or AZI. To further confirm the correlation between Nrf2 activation and the effects of AZI in vivo, we next detected Keap1-Nrf2 signaling pathway in rat lung tissues. Results revealed that Nrf2 was predominantly down-regulated in COPD group and significantly up-regulated in CS + AZI group (Fig. 4C–E). Similar with in vitro data, the expression of Keap1 was not disturbed by CS exposure or AZI treatment compared with sham-treated rats. These results exhibited that Nrf2 was activated in the protective effects of AZI on airway epithelial barrier dysfunction.

### Nrf2 activation plays an essential role in AZI-ameliorating airway epithelial barrier dysfunction

**Fig. 5:**
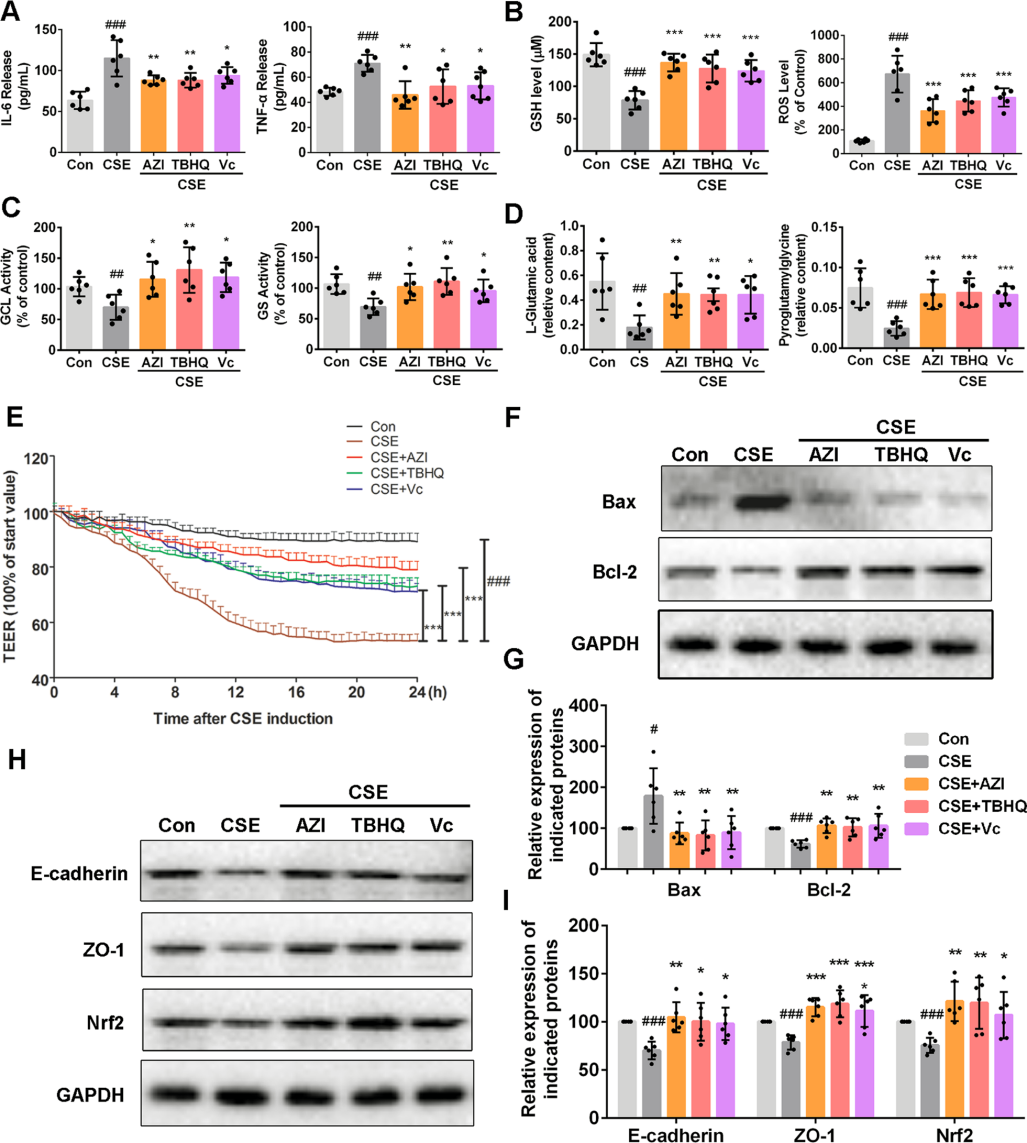
Role of Nrf2 in AZI-ameliorating airway epithelial barrier dysfunction. HBECs were pre-incubated with or without AZI (50 μM), TBHQ (30 μM), Vc (50 μM) for 1 h, and subsequently co-incubated with 3%CSE for 24 h. **A** The levels of IL-6 and TNF-α in the supernatant of HBECs were analyzed by ELISA. **B**, **C** The contents of GSH and ROS, along with the activities of GCL and GS, were measured by relevant assay kits. **D** The contents of L-Glutamic acid and pyroglutamylglycine were measured by LC–MS. **E** TEER was monitored for 24 h at specified time points in HBECs. **F**–**I** Western blot analysis was performed to examine the expressions of indicated proteins in HBECs. Data shown are means ± SD, n = 6. ^#^P < 0.05, ^##^P < 0.01 and ^###^P < 0.001 vs control group; ^*^P < 0.05, ^**^P < 0.01, ^***^P < 0.001 and ns means no significant difference vs CSE group

We next set out to determine whether AZI exerts protection through an Nrf2-dependent mechanism on CS-induced airway epithelial barrier dysfunction. Interestingly, our present results showed that Nrf2 agonist tert-butylhydroquinone (TBHQ, 30 μM) and antioxidant vitamin C (Vc, 50 μM) exhibited the similar effects as AZI (50 μM) on CSE-induced airway epithelial barrier dysfunction in vitro. First, CSE-induced excessive secretions of IL-6 and TNF-α could be inhibited by TBHQ or Vc in HBECs (Fig. 5A). In addition, TBHQ and Vc also significantly reversed the effects of CSE via decreasing ROS level (Fig. 5B) and promoting GSH metabolism, including the up-regulations of GSH, GCL, GS, L-Glutamic acid and pyroglutamylglycine (Fig. 5B–D).We also found that CSE-induced TEER decline was significantly attenuated by TBHQ and Vc, which was similar to AZI treatment (Fig. 5E).

Next, the expressions for apoptosis-related proteins and junction proteins were also examined in HBECs. Our results showed that the decrease of Bax and the increase of Bcl-2, ZO-1 and E-cadherin could also be found in AZI group and reference groups (TBHQ and Vc) compared to CSE group (Fig. 5F–I). Moreover, CSE-induced down-regulation of Nrf2 could also be reversed by TBHQ and Vc (Fig. 5H, I). These results suggested that the protective effects of AZI on airway epithelial barrier dysfunction might be related to Nrf2-mediated antioxidant mechanism.

### Deletion of Nrf2 abrogated the protective effects of AZI in vitro and in vivo

**Fig. 6:**
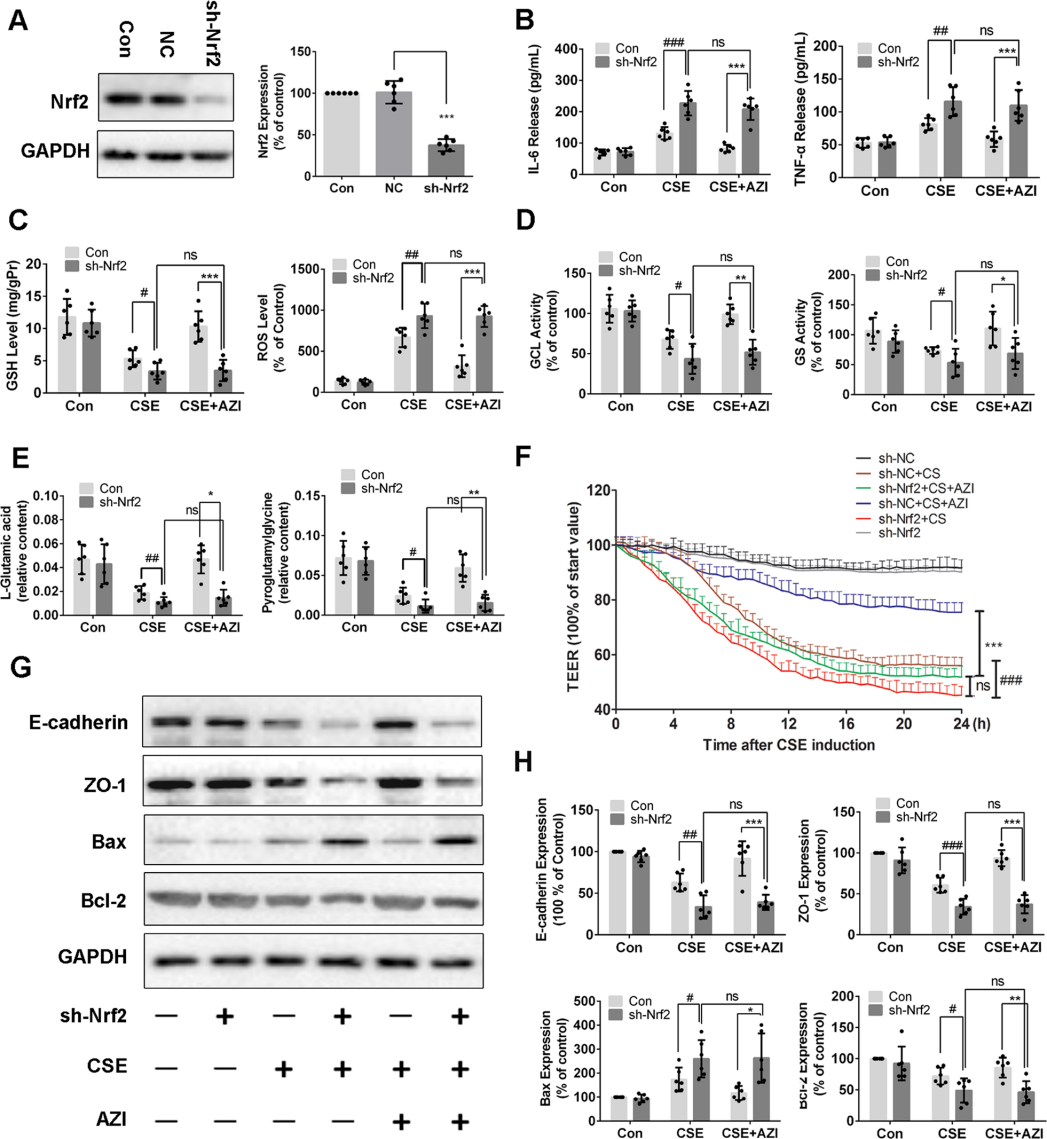
Knockdown of Nrf2 abolished the effects of AZI on airway epithelial barrier dysfunction in vitro. After pre-incubated with or without AZI (50 μM) for 1 h, HBECs with Nrf2 knockdown were co-incubated with 3%CSE for another 24 h. **A** The interference efficiency was determined by Western blot. **B** The levels of IL-6 and TNF-α in the supernatant of HBECs were analyzed by ELISA. **C**, **D** The contents of GSH and ROS along with the activities of GCL and GS were measured by commercial assay kits. **E** The contents of L-Glutamic acid and pyroglutamylglycine were measured by LC–MS. **F** The TEER was monitored for 24 h at specified time points in HBECs. **G**, **H** Western blot analysis was performed to examine the expressions of indicated proteins in HBECs. Data shown are means ± SD, n = 6. ^#^P < 0.05, ^##^P < 0.01, ^###^P < 0.001, ^*^P < 0.05, ^**^P < 0.01 ^***^P < 0.001 and ns means no significant difference, comparison between groups as indicated in the figure

To further confirm the role of Nrf2 in AZI-ameliorating airway epithelial barrier dysfunction, lentiviral-delivered shRNAs was used to knock down Nrf2 expression in HBECs. As demonstrated by western blot in Fig. 6A, Nrf2-shRNA transfection significantly reduced Nrf2 expression (approximately 70%) in HBECs compared with negative control (NC) group. Nrf2 knockdown further aggravated the secretions of IL-6 and TNF-α in CSE-incubated HBECs, while AZI treatment did not exert the protective effects compared with NC cells (Fig. 6B). However, the up-regulations of GSH, L-Glutamic acid, pyroglutamylglycine, GCL and GS caused by AZI were also abolished by sh-Nrf2 transfection in CSE-incubated HBECs (Fig. 6C–E). These results revealed the importance of Nrf2 activation in the restoration of AZI on airway epithelial barrier dysfunction. As expected, Nrf2 knockdown led to further decline of TEER in CSE-exposed cells, and AZI treatment did not reverse the reduction in Nrf2-shRNA-transfected cells compared to NC cells (Fig. 6F). Moreover, deletion of Nrf2 further suppressed the expressions of ZO-1, E-cadherin and Bcl-2, and enhanced the expressions of Bax in CSE-exposed cells. In fact, no significant difference was found in Nrf2-knockdown cells treated with or without AZI treatment under CSE exposure (Fig. 6G, H). These results further confirmed that Nrf2 mediated the effects of AZI on restoring airway epithelial barrier dysfunction caused by CSE.

**Fig. 7:**
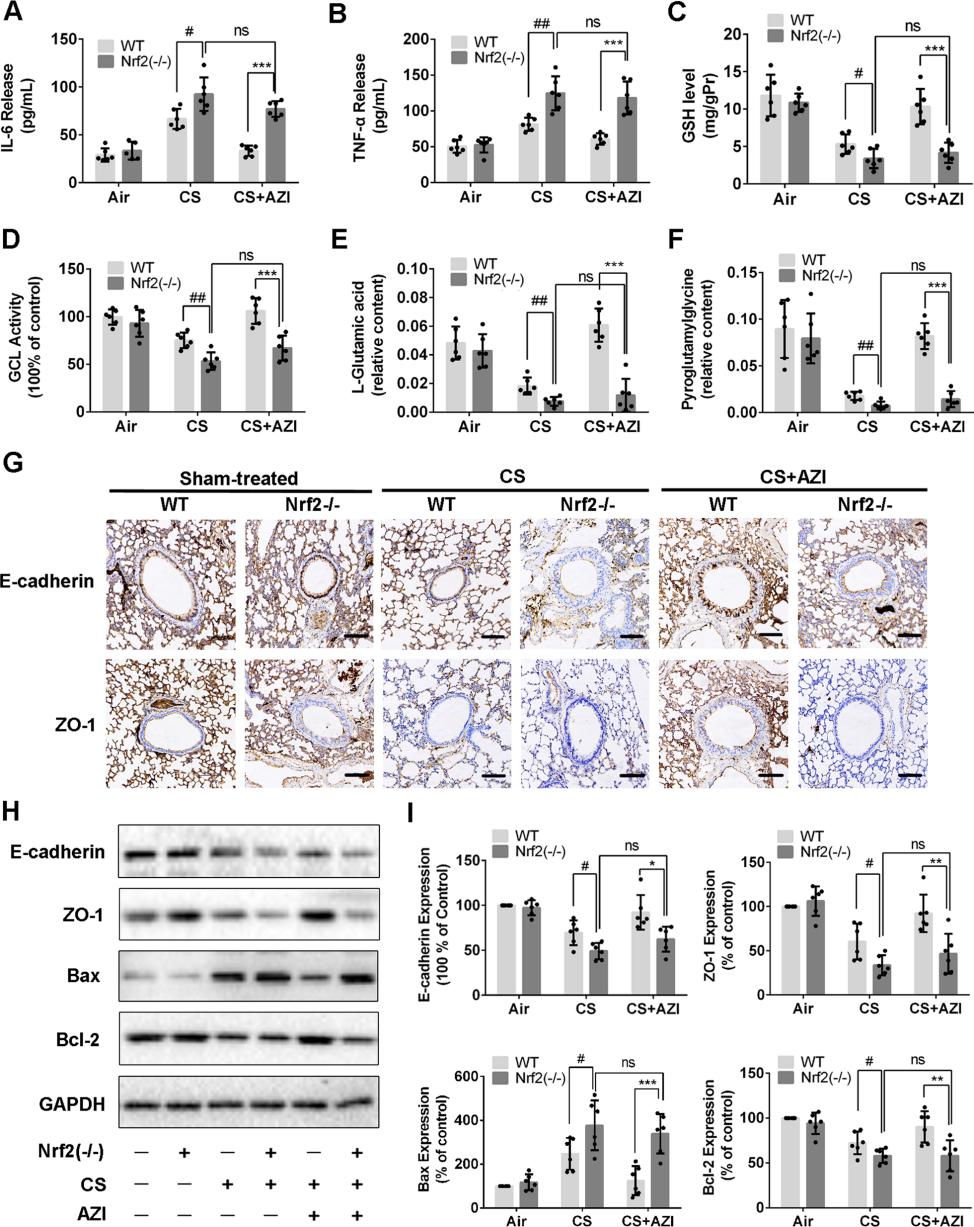
Knockout of Nrf2 deprived the effects of AZI on airway epithelial barrier dysfunction in vivo. **A**, **B** The levels of IL-6 and TNF-α in the BALF of mice were analyzed by ELISA. **C**, **D** The content of GSH and the activity of GCL in lung tissues were measured by relevant assay kits. **E**, **F** The contents of l-Glutamic acid and pyroglutamylglycine in lung tissues were measured by LC–MS. **G** The expressions of E-cadherin and ZO-1in lung tissues were observed by immunohistochemical staining. The scale bar represents 100 μm. **H**, **I** The expressions of proteins relevant to airway epithelial barrier dysfunction were detected by western blot analysis. Data shown are means ± SD, n = 6. ^#^P < 0.05, ^##^P < 0.01, ^*^P < 0.05, ^**^P < 0.01, ^***^P < 0.001 and ns means no significant difference, comparison between groups as indicated in the figure

Nrf2−/− mice susceptibility was used to evaluate the importance of Nrf2 activation in AZI treatment. As shown in Fig. 7A and B, the increased levels of IL-6 and TNF-α were detected in the BALF of Nrf2−/− mice, which was significantly higher than those in WT mice, while AZI failed to inhibit the release of pro-inflammatory cytokines in the absence of Nrf2. Nrf2 knockout also resulted in further down-regulation of GSH, GCL, l-Glutamic acid and pyroglutamylglycine compared to WT mice (Fig. 7C–F). Notably, Nrf2−/− mice showed more obvious airway barrier dysfunction characterized by the abnormal expressions of E-cadherin, ZO-1, Bax and Bcl-2 under CS exposure. Different from the results in WT mice, AZI treatment failed to influence the expressions of the indicated proteins in maintaining airway barrier function in Nrf2−/− mice (Fig. 7G–I). These results further confirmed that AZI ameliorated CS-induced airway barrier dysfunction via activating Nrf2/GCL/GSH signaling pathway in vivo.

### Effects of other macrolide antibiotics on airway epithelial barrier dysfunction induced by CSE

**Fig. 8:**
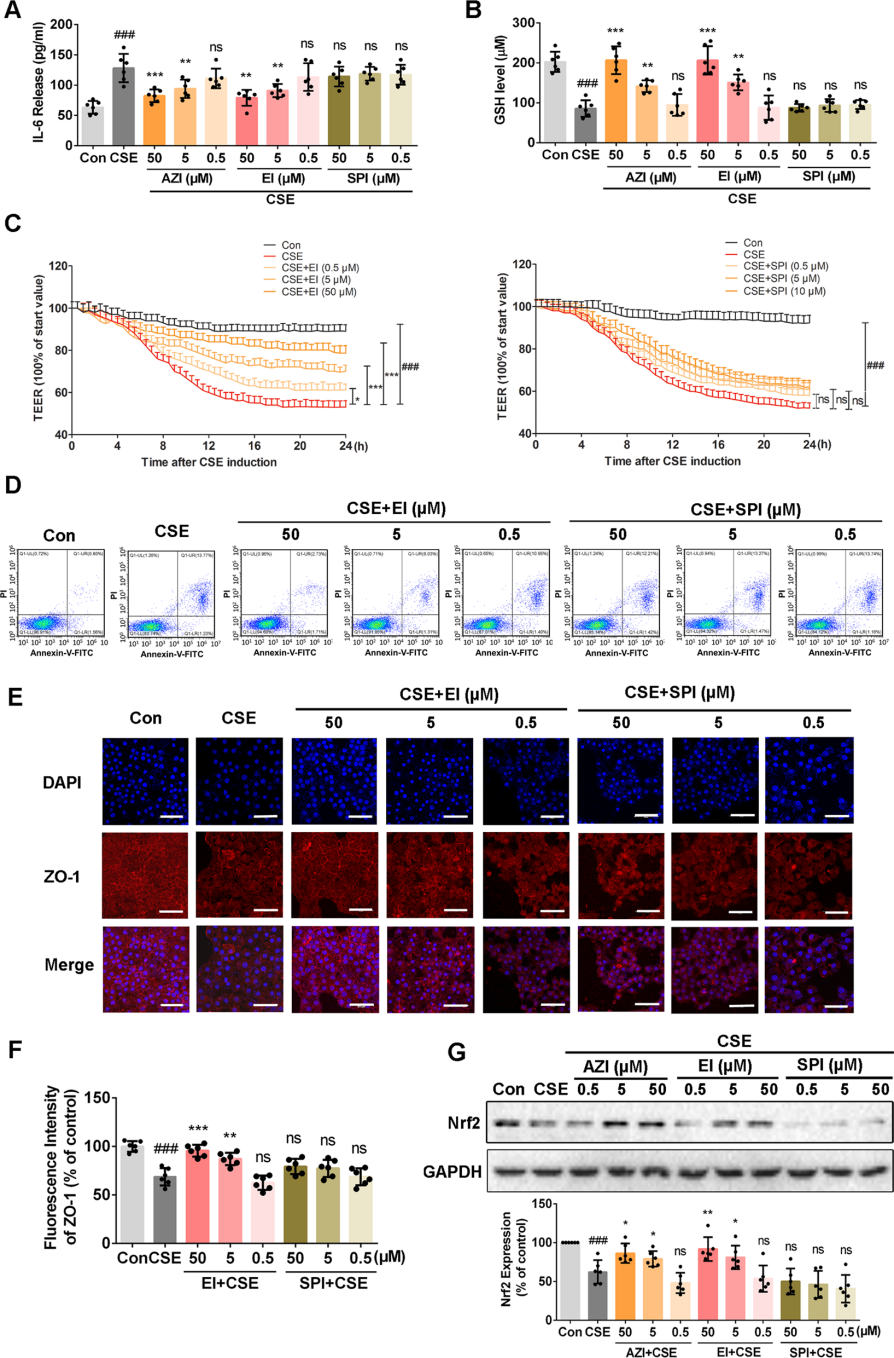
Role of macrolides on airway epithelial barrier dysfunction in CSE-incubated HBECs. HBECs were pre-incubated with or without different concentrations of EI (50, 5, 0.5 μM) or SPI (50, 5, 0.5 μM) for 1 h, and subsequently co-incubated with 3% CSE for 24 h. **A**, **B** The levels of IL-6 and GSH and were measured by relevant commercial assay kits. **C** The TEER was monitored for 24 h at specified time points in HBECs. **D** Flow cytometry assay was used to detect the apoptosis of HBECs. **E**, **F** The expressions of ZO-1 in HBECs were detected by immunofluorescence staining. The scale bar represents 50 μm. **G** The expression of Nrf2 was detected by western blot analysis. Data shown are means ± SD, n = 6. ^##^P < 0.01 and ^###^P < 0.001 vs control group;^*^P < 0.05, ^**^P < 0.01, ^***^P < 0.001 and ns means no significant difference vs CSE group

In order to deeply understand the pharmacological mechanism of AZI, we further analyzed whether other macrolide antibiotics possessed similar pharmacological activities, including erythromycin (EI, 14-membered ring) and spiramycin (SPI, 16-membered ring). We found that EI (5 μM or higher concentration) played a similar role as AZI to inhibit the secretion of IL-6 (Fig. 8A) and restore the decrease of GSH (Fig. 8B) in CSE-exposed HBECs in a concentration-dependent manner, while those pharmacological effects could not be observed in SPI groups. As expected, EI treatment also significantly increased the TEER (Fig. 8C) and enhanced ZO-1 expression (Fig. 8E, F) impaired by CSE incubation, which confirmed that EI could repair the airway epithelial barrier. The results of flow cytometry further revealed that EI could significantly inhibit the apoptosis of airway epithelial cells induced by CSE (Fig. 8D). Finally, similar to AZI, EI could enhance the expression of Nrf2 in airway epithelial cells (Fig. 8G). However, SPI did not exert those pharmacological effects as AZI.

## Discussion

In our present study, we found that AZI not only possessed anti-inflammatory and anti-oxidative properties, but also ameliorated airway epithelial barrier dysfunction by improving TEER and apical junctional complexes. Our study also revealed that AZI significantly up-regulated the expression of Nrf2 to promote GSH metabolism, highlighting the novel role of Nrf2/GCL/GSH signaling pathway in maintaining airway epithelial barrier function. These results demonstrated that AZI prevented CS-induced airway epithelial barrier dysfunction through Nrf2/GCL/GSH signaling pathway.

The bronchial epithelium is responsible for maintaining the airway homeostasis of respiratory system. Destruction of airway barrier integrity exposes sub-epithelial layer to inhaled particles, triggering airway inflammation and immune responses, indicating that airway epithelial barrier dysfunction is closely related with respiratory diseases [5, 9]. It has been confirmed that smoking disrupted apical junctions of airway epithelium, and the reduction of apical junction genes has been observed in the lung tissues of COPD patients [31]. In addition, TJ proteins were also significantly suppressed in lung tissue of patients with end-stage COPD and in air–liquid interface differentiated epithelial cells from these patients [13]. Similarly, our experiments in vitro revealed that CSE exposure caused the degradations of TJ protein ZO-1 and AJ protein E-cadherin with subsequent TEER decline, which eventually leads to airway epithelial barrier dysfunction. It was well known that structural and subsequent functional destruction of epithelial barrier is a typical feature of chronic airway inflammation. Many innate and adaptive immune mediators that may be up-regulated after long-time cigarette smoking, including cytokines, chemokines and apoptosis factors, could regulate the airway epithelium barrier function [5]. As found in our study, CS-increased secretions of pro-inflammatory cytokines and airway epithelium cell apoptosis in vitro and in vivo, which was consistent with above reports.

AZI is commonly indicated for the treatment of respiratory bacterial infection, and exerts immunomodulatory activities in chronic inflammatory disorders, such as COPD [32–34]. Clinically, in patients with severe COPD, continuous therapy of AZI combined with nebulized colistin dramatically prevented the exacerbations of COPD [35]. Preventive administration of AZI reduced the frequency of acute exacerbation and improved the quality of life in COPD patients [20]. However, the underlying mechanism is not completely clear. Here, our study provided evidence that AZI treatment counteracted the CSE-induced TEER reduction and disruption of ZO-1 and E-cadherin, along with the inhibition of inflammatory response and apoptosis in vitro and in vivo. Consistently, a recent study revealed that AZI treatment substantially enhanced epidermal characteristics partially by up-regulation of tight junction proteins in bronchial epithelial cells [36]. In addition, pretreatment of AZI inhibited the secretions of IL-6 and IL-8 in CS-exposed bronchial epithelial cells, suggesting that AZI may be beneficial for smoking-induced airway epithelial barrier dysfunction [37].

Metabolomics is considered as a promising method to accurately determine all low molecular weight metabolites of an organism and reveal its biology and response to pathophysiological stimuli [38]. The results of metabolomics usually contain a continuous stream of high-content information, which will essentially help to understand the differentiating metabolite profiles from a global perspective [39]. Therefore, metabolomics has been widely applied in various fields, such as drug toxicity, disease diagnosis, and pharmacodynamic study [40–42]. To elucidate the mechanisms though which AZI ameliorated CSE-induced airway epithelial barrier dysfunction, we performed metabolomics profiling. Interestingly, our experimental results showed that metabolite set enrichment analysis revealed pathways upregulated by AZI treatment, including GSH metabolism. GSH, the most abundant non-protein thiol compounds in mammalian tissues and cells, is known as the most important endogenous molecule to resist oxidative stress, detoxify xenobiotics and regulate cell proliferation, apoptosis, immune function, and fibrogenesis [43]. Due to the central role of GSH in maintaining cellular redox homeostasis, it is absolutely necessary for a series of biochemical reactions to protect airway epithelial cells from CS-induced oxidative stress [44]. It has been known that CS-induced oxidative stress weakened GSH levels in airway epithelium and disrupted tight junctions, epithelial barrier integrity, finally leading to the impairment of epithelial barrier function [45, 46]. Herein, our study showed that the major molecules or enzymes involved in GSH synthesis and metabolism pathway, including GSH, GCL, GS, GST, l-Glutamic acid and pyroglutamic acid, were all significantly decreased by CSE exposure, while these molecules or enzyme activity were prominently restored by AZI treatment. In fact, AZI has already been reported to suppress ROS release in epithelial cells and prevent oxidative damage in macrophages harvested from 8 transplant recipients [47], indicating the antioxidant potentials of AZI. For the first time to our knowledge, our experimental results highlighted a role for AZI promoting glutathione metabolism in the lungs of CS exposure.

Oxidative stress is highly correlated with the impairment of glutathione metabolism in COPD pathogenesis [48]. GSH synthesis and metabolism are mainly determined by GCL, GS, GST, which are directly regulated by Nrf2 transactivation [49, 50]. Nrf2, a transcription factor significantly expressed in airway epithelial cells, is known to regulate antioxidant and cytoprotective genes through activating antioxidant response elements, showing protective effects on airway epithelium [51]. Previous studies have confirmed that Nrf2 pathway regulates more than 500 genes, including genes that regulate oxidative stress (GCL), inflammation (NF-κB), apoptosis (Bcl-2 and Bax), and autophagy (p62) [52]. It has been confirmed that Nrf2 and some of its target genes constituted a protective signaling pathway against oxidative stress and inflammation in COPD development [53, 54]. And the deficiency of Nrf2 contributes to the pathogenesis of COPD, accompanied with dysregulation of GSH metabolism [49, 51, 55, 56]. A recent observational longitudinal study revealed that GSH was significantly reduced in the blood samples of COPD patients, moreover the expression of Nrf2 in PBMCs were significantly down-regulated in COPD patients at follow-up compared with non-COPD patients [57]. Also, it was reported that activating Nrf2/GCL/GSH antioxidant signaling pathway by quercetin could attenuate toosendanin-induced oxidative stress to prevent hepatotoxicity [58], and Nrf2/GCL/GSH axis could protect mitochondria from methylglyoxal-induced cells [59]. As expected, our results demonstrated that AZI treatment significantly prevented Nrf2 suppression which was induced by CS exposure in vitro and in vivo. Moreover, our experiments further verified the role of Nrf2 by using Nrf2-shRNA, Nrf2 agonists, antioxidant and Nrf2 knockout mice, confirming that the protective effects of AZI on CS-induced airway epithelial barrier dysfunction primarily depends on the activation of Nrf2/GCL/GSH signaling pathway. In addition, our results (in Additional file 4: Fig. S2) further confirmed that, compared with the baseline of control cells, AZI slightly increased the expression of Nrf2, E-cadherin and ZO-1, but the increased value was not statistically different. And AZI also did not affect the levels of IL-6, GCL and GSH in normal HBECs. These results are in accordance with previous reports [60, 61], indicating that AZI would not adversely affect the function and stability of normal cells.

It is also noteworthy that not only AZI, but some other macrolides (e.g. erythromycin, clarithromycin) have also been reported to prevent COPD exacerbations and improve patient quality of life and symptoms [62]. Thus, to further explore whether the protection of airway epithelial barrier is the common pharmacological activity of macrolides, we have repeated the in vitro experiments using erythromycin (EI, 14-membered ring macrolide) and spiramycin (SPI, 16-membered ring macrolide). Data in Fig. 8 revealed that EI could also protect the airway epithelial barrier against CSE and its molecular mechanism was similar to that of AZI, while SPI had no similar pharmacological activity and regulatory effect. In fact, the structure of 16-membered ring macrolide is different from that of 14- and 15-membered ring macrolide, and the bond mode (the hydrogen bond, the hydrophobic bond and the van der Waals force) to the target are also different, which lead to different activity. Pharmacological studies have confirmed that anti-inflammatory and immunomodulatory effects were mainly found in 14- and 15-membered ring macrolides, such as EI and AZI [63], which may explain why SPI showed no obvious effects on CS-induced airway epithelial barrier dysfunction. There are also reports that macrolides with 14- and 15-membered ring, instead of 16-membered ring, have functions other than antibacterial effect: strengthen the epithelial cell barrier and strengthen the tight junction [64]. However, the exact mechanisms of macrolides with 14- and 15-membered ring in protecting the airway epithelial barrier still need to be further studied.

Nevertheless, there are some limitations in this study: (1) We only investigated the preventive effect of AZI, but its clinical therapeutic effect on protecting airway epithelial barrier is still worthy of further verification; (2) We have already found that Nrf2 is the key mediator in the protective effect of AZI on airway epithelial barrier, but how AZI activates Nrf2 is still unknown, which is our next research objective; (3) Different pharmacological effects of macrolides on airway epithelial barrier may be related to the difference of structural characteristics, which requires molecular docking and structure modeling methods to further clarify the underlying mechanisms.

## Conclusion

Our current data from multiple models, including PBECs, HBECs, SD rats as well as Nrf2−/− mice reveal the preventive effects of AZI on CSE-induced airway epithelial barrier dysfunction in COPD and underscore the importance of Nrf2/GCL/GSH antioxidant signaling pathway as the regulatory target of macrolides in the protection of airway epithelial barrier. Our findings expand new insights into the molecular mechanisms by which AZI restores CS-induced airway epithelial barrier dysfunction during COPD pathogenesis, providing experimental evidence for optimizing treatment strategies of COPD and target-based discovery and development of novel macrolides.

## Supplementary Information

**Additional file 1: Fig. S1.** Identification of primary bronchial epithelial cells primary bronchial epithelial cells (PBECs).

**Additional file 2: Fig S2.** Effects of AZI treatment alone on HBECs.

**Additional file 3: Table S1.** Characteristics of airway brush specimen subjects (n=6).

**Additional file 4: Table S2.** Differential expressed metabolites involved in MSEA.

## Data Availability

The datasets used and/or analysed during the current study are available from the corresponding author on reasonable request.

## Abbreviations

AJ: Adherent junction
AZI: Azithromycin
BALF: Bronchoalveolar lavage fluid
COPD: Chronic obstructive pulmonary disease
CS: Cigarette smoke
CSE: Cigarette smoke extract
GCL: Glutamate cysteine ligase
GS: Glutathione synthetase
GST: Glutathione S-transferase
HBECs: Human bronchial epithelial cells
Keap1: Kelch-like ECH-associated protein-1
MSEA: Metabolite set enrichment analysis
NC: Negative control
Nrf2: Nuclear factor erythroid 2-related factor 2
PBECs: Primary bronchial epithelial cells
PCA: Principal component analysis
SD: Sprague Dawley
shRNAs: Short-hairpin RNAs
TBHQ: Tert-butylhydroquinone
TEER: Transepithelial electronic resistance
TJ: Tight junction
TNF-α: Tumor necrosis factor-alpha
Vc: Vitamin C
ZO-1: Zonula occludens-1
