# Supplementary material for: Azithromycin ameliorated cigarette smoke-induced airway epithelial barrier dysfunction by activating Nrf2/GCL/GSH signaling pathway
Source: Respir Res. 2023 Mar 6;24:69. doi: 10.1186/s12931-023-02375-9 (PMC9990325; doi:10.1186/s12931-023-02375-9)
Supplement: Supplementary file 1 — Additional file 1: Fig. S1. Identification of primary bronchial epithelial cells primary bronchial epithelial cells (PBECs). [file 12931_2023_2375_MOESM1_ESM.pdf]

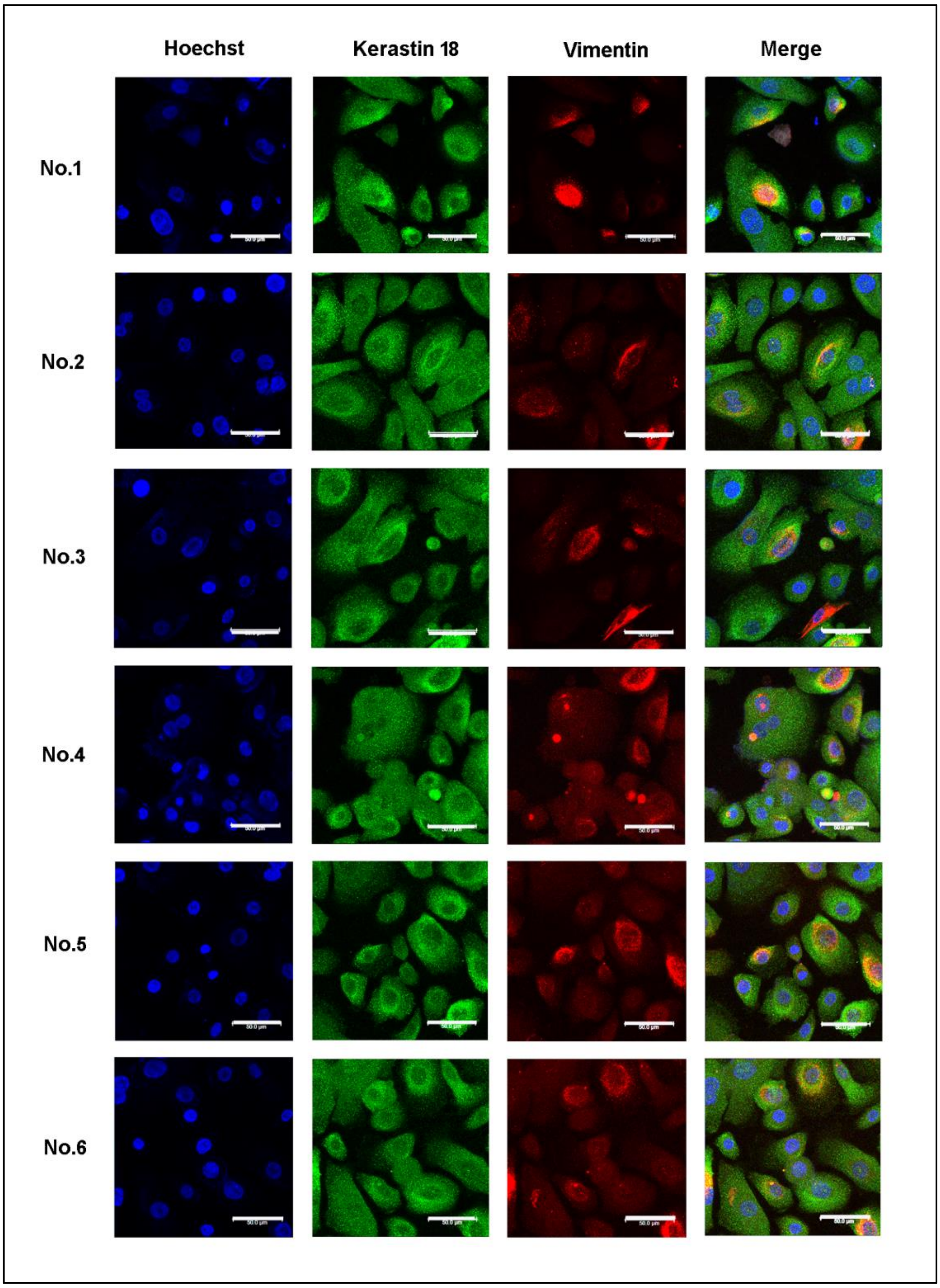

Fig S1. Identification of primary bronchial epithelial cells (PBECs). Immunofluorescence staining was performed to identify PBECs from healthy subjects (n=6). The scale bar represents 50 μm. anti-Keratin18 (#4548T) and anti-Vimentin (#5741T) primary antibodies, along with Alexa Fluor 594/488 conjugated anti-rabbit (#8889S)/mouse (#4408S) IgG (H+L) F(ab')2 Fragments

were purchased from Cell Signaling Technology (Danvers, MA, USA). The dilution ratio of primary antibodies (Keratin18 and Vimentin) was 1:100. Hoechst (#C1027) was purchased from Beyotime Biotechnology (Shanghai, China).
