# Supplementary material for: Azithromycin ameliorated cigarette smoke-induced airway epithelial barrier dysfunction by activating Nrf2/GCL/GSH signaling pathway
Source: Respir Res. 2023 Mar 6;24:69. doi: 10.1186/s12931-023-02375-9 (PMC9990325; doi:10.1186/s12931-023-02375-9)
Supplement: Supplementary file 2 — Additional file 2: Fig S2. Effects of AZI treatment alone on HBECs. [file 12931_2023_2375_MOESM2_ESM.pdf]

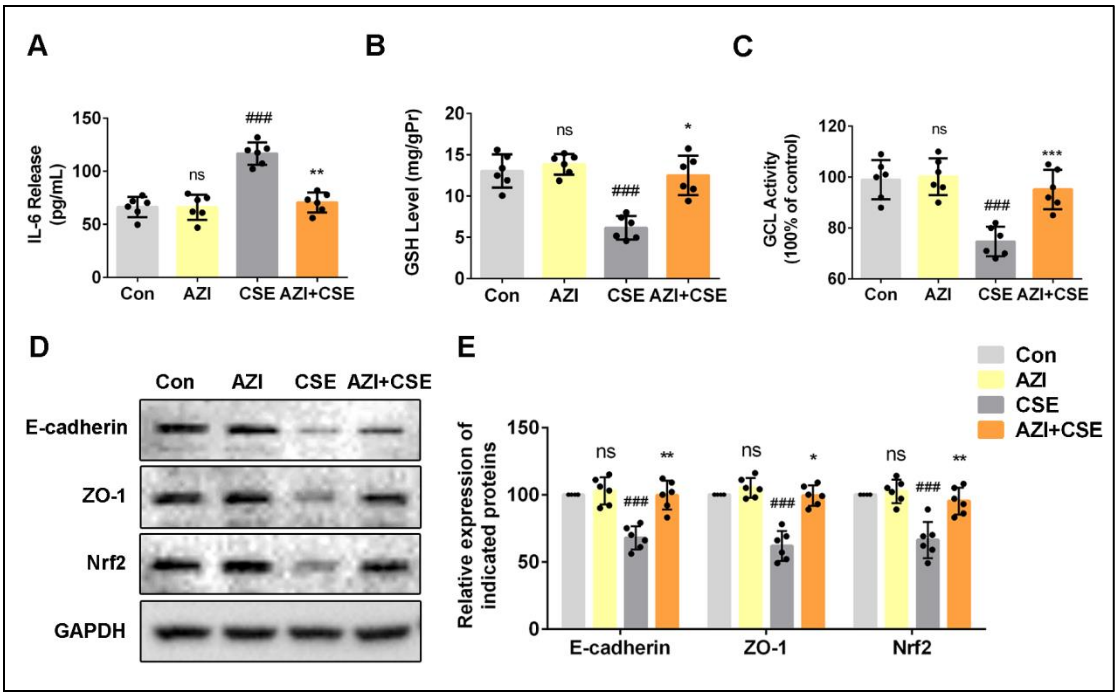

Fig S2 Effects of AZI treatment alone on HBECs. HBECs were pre-incubated with or without AZI (50 μM) for 1 h, and subsequently co-incubated with 0% or 3% CSE for 24 h. (A, B) The level of IL-6 in the supernatant of HBECs was measured by ELISA. (B, C) The content of GSH and the activity of GCL were measured by commercial assay kits. (D, E) Western blot was performed to examine the expressions of E-cadherin, ZO-1 and Nrf2 in HBECs. Data shown are means ± SD, n = 6. $^{###}P < 0.001$ and ns means no significant difference vs control group; $^{*}P < 0.05$, $^{**}P < 0.01$, $^{***}P < 0.001$ vs CSE group.
