# Supplementary material for: Azithromycin ameliorated cigarette smoke-induced airway epithelial barrier dysfunction by activating Nrf2/GCL/GSH signaling pathway
Source: Respir Res. 2023 Mar 6;24:69. doi: 10.1186/s12931-023-02375-9 (PMC9990325; doi:10.1186/s12931-023-02375-9)
Supplement: Supplementary file 3 — Additional file 3: Table S1. Characteristics of airway brush specimen subjects (n=6). [file 12931_2023_2375_MOESM3_ESM.docx]

**Table S1 Characteristics of airway brush specimen subjects (n=6)**

| **Subject** | **Age (yrs)** | **Sex** | **Smoking history** | **FEV_1_/FVC (%)** |
| --- | --- | --- | --- | --- |
| 1 | 32 | M | No | 92 |
| 2 | 40 | M | No | 87 |
| 3 | 22 | M | No | 90 |
| 4 | 26 | F | No | 85 |
| 5 | 31 | F | No | 81 |
| 6 | 25 | F | No | 82 |

FEV_1_: forced expiratory volume in the first one second; FVC: forced vital capacity
