# Supplementary material for: Azithromycin ameliorated cigarette smoke-induced airway epithelial barrier dysfunction by activating Nrf2/GCL/GSH signaling pathway
Source: Respir Res. 2023 Mar 6;24:69. doi: 10.1186/s12931-023-02375-9 (PMC9990325; doi:10.1186/s12931-023-02375-9)
Supplement: Supplementary file 4 — Additional file 4: Table S2. Differential expressed metabolites involved in MSEA. [file 12931_2023_2375_MOESM4_ESM.docx]

**Table S2 Differential expressed metabolites involved in MSEA**

| **No.** | **Name** | **Formula** | **Type** | ***m/z*** |
| --- | --- | --- | --- | --- |
| 1 | Pyroglutamic acid | C_5_H_7_NO_3_ | [M-H]^-^ | 128.0351 |
| 2 | Pyroglutamylglycine | C_7_H_10_N_2_O_4_ | [M-H]^-^ | 485.0588 |
| 3 | S-butyl-DL-homocysteine (S,R)-sulfoximine | C_11_H_14_N_2_O_3_ | [M-H]^-^ | 221.0931 |
| 4 | Serylvalylglycylglutamic acid | C_15_H_26_N_4_O_8_ | [M-H]^-^ | 389.1676 |
| 5 | S-geranylgeranyl-L-cysteine | C_19_H_40_NO_5_P | [M-H]^-^ | 392.2583 |
| 6 | Sulfolithocholylglycine | C_26_H_43_NO_7_S | [M+H]^+^ | 514.2836 |
| 7 | L-Glutamic acid | C_5_H_9_NO_4_ | [M+H]^+^ | 148.0516 |
| 8 | S-Benzyl-L-cysteine sulfoxide | C_10_H_13_NO_3_S | [M+H]^+^ | 228.0682 |
| 9 | gamma-Glutamyl-S-methylcysteine | C_9_H_16_N_2_O_5_S | [M+H]^+^ | 265.0817 |
| 10 | N-[(3a,5b,7a,12a)-3,12-dihydroxy-24-oxo-7-  (sulfooxy)cholan-24-yl]-Glycine | C_26_H_43_NO_9_S | [M+H]^+^ | 546.2718 |
| 11 | N-[(Cyclohexylamino)Carbonyl]Glycine | C_9_H_16_N_2_O_3_ | [M+H]^+^ | 201.1208 |
| 12 | D-Pantothenoyl-L-cysteine | C_12_H_22_N_2_O_6_S | [M+H]^+^ | 323.1308 |
| 13 | Palmitoylglycine | C_18_H_35_NO_3_ | [M-H]^-^ | 312.2528 |
| 14 | N-Lauroylglycine | C_14_H_27_NO_3_ | [M-H]^-^ | 256.1906 |
| 15 | Myristoylglycine | C_16_H_31_NO_3_ | [M-H]^-^ | 284.2219 |
| 16 | Pentacosanoylglycine | C_27_H_53_NO_3_ | [M+H]^+^ | 440.411 |
| 17 | N-Nonanoylglycine | C_11_H_21_NO_3_ | [M+H]^+^ | 216.16 |
| 18 | S-Adenosylhomocysteine | C_14_H_20_N_6_O_5_S | [M+H]^+^ | 385.131 |
| 19 | hydrogen N- (1-oxododecyl)-L-glutamic acid | C_17_H_31_NO_5_ | [M+H]^+^ | 330.2285 |
| 20 | N-Acetylaspartylglutamic acid | C_11_H_16_N_2_O_8_ | [M+H]^+^ | 305.0985 |
| 21 | N-[(4Z,7Z,10Z,13Z,16Z,19Z)-docosahexaenoyl]-L-glutamic acid | C_27_H_39_NO_5_ | [M+H]^+^ | 458.2931 |
| 22 | N-Pyridoxyl-2-Methyl-L-Glutamic acid -5'-Monophosphate | C_14_H_21_N_2_O_9_P | [M+H]^+^ | 393.1062 |
| 23 | 2,3-dipalmitoyl-S-glycerylcysteine | C_38_H_73_NO_6_S | [M+H]^+^ | 672.5342 |

| **No.** | | **Name** | **con-1** | **con-2** | | **con-3** | | **con-4** | | **con-5** | | **con-6** | |
| --- | --- | --- | --- | --- | --- | --- | --- | --- | --- | --- | --- | --- | --- |
| 1 | Pyroglutamic acid | | 0.094499 | | 0.090103 | | 0.058815 | | 0.066805 | | 0.1044 | | 0.089594 |
| 2 | Pyroglutamylglycine | | 0.092068 | | 0.170985 | | 0.055965 | | 0.068486 | | 0.102114 | | 0.127431 |
| 3 | S-butyl-DL-homocysteine (S,R)-  sulfoximine | | 0.096789 | | 0.215911 | | 0.011199 | | 0.088595 | | 0.077796 | | 0.174846 |
| 4 | Serylvalylglycylglutamic acid | | 0.005231 | | 0.010106 | | 0.004601 | | 0.011269 | | 0.006242 | | 0.011394 |
| 5 | S-geranylgeranyl-L-cysteine | | 0.442558 | | 0.557064 | | 0.308265 | | 0.161425 | | 0.694426 | | 0.592062 |
| 6 | Sulfolithocholylglycine | | 1.332544 | | 1.284973 | | 1.199339 | | 0.849306 | | 0.933232 | | 1.247909 |
| 7 | L-Glutamic acid | | 0.097974 | | 0.021985 | | 0.075297 | | 0.046546 | | 0.04836 | | 0.059675 |
| 8 | S-Benzyl-L-cysteine sulfoxide | | 0.192817 | | 0.188691 | | 0.186085 | | 0.100263 | | 0.133571 | | 0.182917 |
| 9 | gamma-Glutamyl-S-  methylcysteine | | 0.008432 | | 0.010448 | | 0.010974 | | 0.017744 | | 0.00952 | | 0.021971 |
| 10 | N-[(3a,5b,7a,12a)-3,12-  dihydroxy-24-oxo-7-  (sulfooxy)cholan-24-yl]-  Glycine | | 1.247826 | | 1.200353 | | 0.692286 | | 0.135622 | | 0.655217 | | 1.092311 |
| 11 | N-[(Cyclohexylamino)Carbonyl]  Glycine | | 0.156032 | | 0.126057 | | 0.090492 | | 0.06614 | | 0.087744 | | 0.13479 |
| 12 | D-Pantothenoyl-L-cysteine | | 0.041636 | | 0.031953 | | 0.005768 | | 0.007976 | | 0.017466 | | 0.041414 |
| 13 | Palmitoylglycine | | 0.020894 | | 0.040184 | | 0.026277 | | 0.030444 | | 0.031936 | | 0.031177 |
| 14 | N-Lauroylglycine | | 0.007679 | | 0.127196 | | 0.011577 | | 0.008268 | | 0.014834 | | 0.004472 |
| 15 | Myristoylglycine | | 0.006343 | | 0.075525 | | 0.00579 | | 0.006731 | | 0.0105 | | 0.003194 |
| 16 | Pentacosanoylglycine | | 0.025636 | | 0.027307 | | 0.023778 | | 0.009616 | | 0.017267 | | 0.022084 |
| 17 | N-Nonanoylglycine | | 0.152035 | | 0.165787 | | 0.140465 | | 0.088757 | | 0.109409 | | 0.18715 |
| 18 | S-Adenosylhomocysteine | | 0.061972 | | 0.021767 | | 0.081648 | | 0.080351 | | 0.049148 | | 0.084914 |
| 19 | hydrogen N- (1-oxododecyl)-L-  glutamic acid | | 0.078051 | | 0.074492 | | 0.071358 | | 0.055015 | | 0.055783 | | 0.096822 |
| 20 | N-Acetylaspartylglutamic acid | | 0.001446 | | 0.001006 | | 0.001835 | | 0.000839 | | 0.001025 | | 0.001142 |
| 21 | N-[(4Z,7Z,10Z,13Z,16Z,19Z)-  docosahexaenoyl]-L-glutamic  acid | | 0.716161 | | 0.664109 | | 0.596884 | | 0.379754 | | 0.471382 | | 0.667572 |
| 22 | N-Pyridoxyl-2-Methyl-L-Glutamic acid -5'-Monophosphate | | 0.089404 | | 0.098393 | | 0.532302 | | 0.62525 | | 0.26907 | | 0.077834 |
| 23 | 2,3-dipalmitoyl-S-  glycerylcysteine | | 0.034978 | | 0.021464 | | 0.004732 | | 0.005138 | | 0.013263 | | 0.007318 |

| **No.** | | **Name** | **COPD-1** | **COPD-2** | | **COPD-3** | | **COPD-4** | | **COPD-5** | | **COPD-6** | |
| --- | --- | --- | --- | --- | --- | --- | --- | --- | --- | --- | --- | --- | --- |
| 1 | Pyroglutamic acid | | 0.011898 | | 0.033157 | | 0.024858 | | 0.025245 | | 0.027769 | | 0.047581 |
| 2 | Pyroglutamylglycine | | 0.012685 | | 0.021312 | | 0.027019 | | 0.012505 | | 0.013755 | | 0.031062 |
| 3 | S-butyl-DL-homocysteine (S,R)-  sulfoximine | | 0.00351 | | 0.019857 | | 0.018898 | | 0.005893 | | 0.006482 | | 0.020487 |
| 4 | Serylvalylglycylglutamic acid | | 0 | | 0 | | 0 | | 0 | | 0 | | 0 |
| 5 | S-geranylgeranyl-L-cysteine | | 0.153705 | | 0.274908 | | 0.413361 | | 0.332502 | | 0.365753 | | 0.516696 |
| 6 | Sulfolithocholylglycine | | 0.77127 | | 0.982652 | | 1.127259 | | 0.975169 | | 1.072686 | | 1.221225 |
| 7 | L-Glutamic acid | | 0.018436 | | 0.027069 | | 0.023508 | | 0.023169 | | 0.025485 | | 0.051975 |
| 8 | S-Benzyl-L-cysteine sulfoxide | | 0.108068 | | 0.13458 | | 0.15676 | | 0.140932 | | 0.155025 | | 0.196487 |
| 9 | gamma-Glutamyl-S-  methylcysteine | | 0.015986 | | 0.02236 | | 0.020797 | | 0.020785 | | 0.022864 | | 0.02266 |
| 10 | N-[(3a,5b,7a,12a)-3,12-  dihydroxy-24-oxo-7-  (sulfooxy)cholan-24-yl]-  Glycine | | 0.415276 | | 0.715413 | | 0.435685 | | 0.510008 | | 0.561008 | | 1.173079 |
| 11 | N-[(Cyclohexylamino)Carbonyl]  Glycine | | 0.047773 | | 0.066481 | | 0.067453 | | 0.057156 | | 0.062872 | | 0.084979 |
| 12 | D-Pantothenoyl-L-cysteine | | 0.002547 | | 0.002714 | | 0.004247 | | 0.003228 | | 0.003551 | | 0.012952 |
| 13 | Palmitoylglycine | | 0.007534 | | 0.012901 | | 0.010162 | | 0.007633 | | 0.008397 | | 0.021562 |
| 14 | N-Lauroylglycine | | 0.015307 | | 0.005496 | | 0.007092 | | 0.003155 | | 0.003471 | | 0.005979 |
| 15 | Myristoylglycine | | 0.008732 | | 0.003631 | | 0.005406 | | 0.000944 | | 0.001038 | | 0.002693 |
| 16 | Pentacosanoylglycine | | 0.012258 | | 0.015454 | | 0.020564 | | 0.013015 | | 0.014317 | | 0.025041 |
| 17 | N-Nonanoylglycine | | 0.068356 | | 0.08927 | | 0.084264 | | 0.09989 | | 0.109879 | | 0.153789 |
| 18 | S-Adenosylhomocysteine | | 0.036718 | | 0.062524 | | 0.041101 | | 0.043246 | | 0.047571 | | 0.069659 |
| 19 | hydrogen N- (1-oxododecyl)-L-  glutamic acid | | 0.052021 | | 0.065863 | | 0.075128 | | 0.067094 | | 0.073803 | | 0.09728 |
| 20 | N-Acetylaspartylglutamic acid | | 0.00084 | | 0 | | 0.002733 | | 0.000627 | | 0.00069 | | 0.001621 |
| 21 | N-[(4Z,7Z,10Z,13Z,16Z,19Z)-  docosahexaenoyl]-L-glutamic  acid | | 0.297433 | | 0.411951 | | 0.434693 | | 0.34309 | | 0.377399 | | 0.511629 |
| 22 | N-Pyridoxyl-2-Methyl-L-Glutamic acid -5'-Monophosphate | | 0.032538 | | 0.050418 | | 0.023093 | | 0.056641 | | 0.062305 | | 0.128487 |
| 23 | 2,3-dipalmitoyl-S-  glycerylcysteine | | 0.003154 | | 0.007138 | | 0.002591 | | 0.002888 | | 0.003177 | | 0.00225 |

| **No.** | | **Name** | **CS+AZI-1** | **CS+AZI-2** | | **CS+AZI-3** | | **CS+AZI-4** | | **CS+AZI-5** | | **CS+AZI-6** | |
| --- | --- | --- | --- | --- | --- | --- | --- | --- | --- | --- | --- | --- | --- |
| 1 | Pyroglutamic acid | | 0.155721 | | 0.192285 | | 0.173961 | | 0.170248 | | 0.184021 | | 0.237536 |
| 2 | Pyroglutamylglycine | | 0.072653 | | 0.096282 | | 0.094929 | | 0.090156 | | 0.083773 | | 0.114087 |
| 3 | S-butyl-DL-homocysteine (S,R)-  sulfoximine | | 0.087616 | | 0.098708 | | 0.116003 | | 0.112743 | | 0.14152 | | 0.084174 |
| 4 | Serylvalylglycylglutamic acid | | 0.01815 | | 0.016982 | | 0.036345 | | 0.019274 | | 0.016166 | | 0.021978 |
| 5 | S-geranylgeranyl-L-cysteine | | 0 | | 0 | | 0 | | 0 | | 0 | | 0 |
| 6 | Sulfolithocholylglycine | | 0.464453 | | 0.614944 | | 0.624606 | | 0.618261 | | 0.543797 | | 0.571852 |
| 7 | L-Glutamic acid | | 0.055446 | | 0.072544 | | 0.086423 | | 0.062818 | | 0.071056 | | 0.066601 |
| 8 | S-Benzyl-L-cysteine sulfoxide | | 0.061344 | | 0.08138 | | 0.085445 | | 0.078549 | | 0.057634 | | 0.063646 |
| 9 | gamma-Glutamyl-S-  methylcysteine | | 0.027458 | | 0.035854 | | 0.038284 | | 0.035694 | | 0.034312 | | 0.027676 |
| 10 | N-[(3a,5b,7a,12a)-3,12-  dihydroxy-24-oxo-7-  (sulfooxy)cholan-24-yl]-  Glycine | | 0 | | 0 | | 0 | | 0 | | 0 | | 0 |
| 11 | N-[(Cyclohexylamino)Carbonyl]  Glycine | | 0.144517 | | 0.123546 | | 0.178673 | | 0.275851 | | 0.212223 | | 0.173132 |
| 12 | D-Pantothenoyl-L-cysteine | | 0.028468 | | 0.024548 | | 0.058938 | | 0.030387 | | 0.03715 | | 0.02353 |
| 13 | Palmitoylglycine | | 0.032973 | | 0.043549 | | 0.076374 | | 0.042859 | | 0.043624 | | 0.080699 |
| 14 | N-Lauroylglycine | | 0.03655 | | 0.040407 | | 0.107803 | | 0.131492 | | 0.125017 | | 0.202875 |
| 15 | Myristoylglycine | | 0.016003 | | 0.020823 | | 0.055614 | | 0.053354 | | 0.055487 | | 0.099366 |
| 16 | Pentacosanoylglycine | | 0.020729 | | 0.028564 | | 0.024032 | | 0.030319 | | 0.027624 | | 0.020276 |
| 17 | N-Nonanoylglycine | | 0.120946 | | 0.203189 | | 0.148254 | | 0.132339 | | 0.157286 | | 0.163259 |
| 18 | S-Adenosylhomocysteine | | 0.061027 | | 0.098478 | | 0.075768 | | 0.069862 | | 0.058477 | | 0.068359 |
| 19 | hydrogen N- (1-oxododecyl)-L-  glutamic acid | | 0.06891 | | 0.090367 | | 0.089282 | | 0.095991 | | 0.09668 | | 0.107002 |
| 20 | N-Acetylaspartylglutamic acid | | 0.013092 | | 0.005472 | | 0.013707 | | 0.033187 | | 0.004213 | | 0.001685 |
| 21 | N-[(4Z,7Z,10Z,13Z,16Z,19Z)-  docosahexaenoyl]-L-glutamic  acid | | 0.244795 | | 0.285688 | | 0.322528 | | 0.370966 | | 0.400732 | | 0.294361 |
| 22 | N-Pyridoxyl-2-Methyl-L-Glutamic acid -5'-Monophosphate | | 0.02944 | | 0.040212 | | 0.03982 | | 0.03773 | | 0.036078 | | 0.042489 |
| 23 | 2,3-dipalmitoyl-S-  glycerylcysteine | | 0.006009 | | 0.007632 | | 0.011983 | | 0.004422 | | 0 | | 0.001431 |
